# Static test flakiness prediction: How Far Can We Go?

**DOI:** 10.1007/s10664-022-10227-1

**Published:** 2022-10-01

**Authors:** Valeria Pontillo, Fabio Palomba, Filomena Ferrucci

**Affiliations:** Software Engineering (SeSa) Lab - Department of Computer Science, University of Salerno, Fisciano, Italy

**Keywords:** Flaky tests, Software testing, Machine learning

## Abstract

Test flakiness is a phenomenon occurring when a test case is non-deterministic and exhibits both a passing and failing behavior when run against the same code. Over the last years, the problem has been closely investigated by researchers and practitioners, who all have shown its relevance in practice. The software engineering research community has been working toward defining approaches for detecting and addressing test flakiness. Despite being quite accurate, most of these approaches rely on expensive dynamic steps, e.g., the computation of code coverage information. Consequently, they might suffer from scalability issues that possibly preclude their practical use. This limitation has been recently targeted through machine learning solutions that could predict the flakiness of tests using various features, like source code vocabulary or a mixture of static and dynamic metrics computed on individual snapshots of the system. In this paper, we aim to perform a step forward and predict test flakiness *only using static metrics*. We propose a large-scale experiment on 70 Java projects coming from the iDFlakies and FlakeFlagger datasets. First, we statistically assess the differences between flaky and non-flaky tests in terms of 25 test and production code metrics and smells, analyzing both their individual and combined effects. Based on the results achieved, we experiment with a machine learning approach that predicts test flakiness solely based on static features, comparing it with two state-of-the-art approaches. The key results of the study show that the static approach has performance comparable to those of the baselines. In addition, we found that the characteristics of the production code might impact the performance of the flaky test prediction models.

## Introduction

Regression testing is a widely used approach to verify whether newly committed code changes introduce software faults (Pezze and Young 2008). Developers rely on test cases to decide on whether to merge pull requests or even deploy the entire system (Grano et al. 2020). Perhaps more importantly, developer’s productivity is partially dependent on the outcome of test cases (Catolino et al. 2019; Micco 2017): this is mainly due to their ability to identify real faults in a timely and reliable fashion (Perez et al. 2017).

Unfortunately, even tests can be affected by defects and, sometimes, they can suffer from the so-called *flakiness* (Luo et al. 2014): this happens when a test exhibits both a passing and failing behavior when run against the same code, being therefore unreliable and producing a non-deterministic outcome. While the amount of flaky tests in software systems is typically limited - according to previous literature on the matter (Eck et al. 2019; Fowler 2011; Micco 2017), flakiness explicitly arises in around 2% of the tests. Nonetheless, it is hard to precisely estimate the amount of flaky tests because of their intrinsic non-determinism, i.e., tests might be flaky even though their flakiness does not arise. This is why researchers advocated the need of considering *all tests as potentially flaky* (Cordy et al. 2022; Harman and O’Hearn 2018). At the same time, flaky tests have a profound impact on testing activities: (1) They may hide real defects and be hard to reproduce because of their non-determinism (Luo et al. 2014); (2) They increase testing costs, as developers invest time debugging failures that are not real (Lacoste 2009); and (3) They can reduce the overall developer’s confidence on test cases, potentially leading to neglect real defects (Eck et al. 2019). In addition, the presence of flaky tests might impact a number of collateral testing tools. In mutation testing, the mutation score might lead to variations due to flakiness, confounding this variability with the influence of the quality of the test that the mutation score aims at addressing (Cordy et al. 2022). Still, in automated program repair, the certainty that a repair is correct may be affected by flaky tests, other than possibly making the repair technique unable to localize the point where to attempt a patch (Cordy et al. 2022). The potential harms of test code flakiness have been made more and more popular by practitioners and companies worldwide (e.g., (Fowler 2011; Micco 2017)), who all called for automated mechanisms to detect and deal with it.

The software engineering research community has been contributing to the body of knowledge through empirical investigations aiming at eliciting the causes of flakiness (Eck et al. 2019; Lam et al. 2020; Lam et al. 2020; Luo et al. 2014; Memon and Cohen 2013) as well as with the definition of techniques for detecting and addressing them (Bell et al. 2018; Daniel et al. 2009; Terragni et al. 2020; Zhang et al. 2014). Despite the promising results achieved so far, most of the identification techniques require test cases to be re-run multiple times: for instance, the most well-known approach is called ReRun and consists of executing the same test *N* times, with *N* being a variable that goes from dozens to hundreds of executions. As the reader may understand, the poor scalability of ReRun makes it often unusable in practice; in addition, there is no guarantee to discover the flakiness over the *N* runs.

To face this limitation, researchers devised alternatives like DeFlaker (Bell et al. 2018), that works at commit-level and relies on the differential code coverage extracted from the analysis of a test execution from a commit to another. In a complementary manner, the use of machine learning approaches has been proposed. Pinto et al. (Pinto et al. 2020) and further replications (Camara et al. 2021b; Haben et al. 2021) exploited the test code dictionary to discriminate the presence of potential flakiness. More recently, Alshammari et al. (2021) devised a supervised learning model that, using a mixture of code and coverage metrics, can predict flaky tests with an accuracy up to 86%. While these previous research efforts have shown promising results, they all involve steps that might deteriorate the scalability of the proposed techniques. More particularly, the techniques proposed by Bell et al. (Bell et al. 2018) and Alshammari et al. (Alshammari et al. 2021) require the computation of dynamic features, while the approach by Pinto et al. (Pinto et al. 2020) relies on natural language processing, which is known to be costly as the corpus of the text to analyze increases in size (Banko and Brill 2001).

To face the scalability limitations of the currently available techniques, our previous work (Pontillo et al. 2021) aimed at conducting a feasibility study to assess whether a static prediction of test flakiness would be possible, i.e., whether we could identify likely flaky test cases only based on their design. In particular, we took into account the iDFlakies dataset,^1^ and investigated the differences between flaky and non-flaky tests in terms of 25 test and production code metrics and smells. We first studied the distribution of these indicators individually, observing that a number of metrics and smells are more likely to be observed on flaky tests. Then, we also considered the combined effects of the indicators by computing a logistic regression model relating them to test flakiness: also in this case, the results showed the presence of static indicators that are statistically connected to flakiness.

The promising results achieved by our previous work (Pontillo et al. 2021) indicated the feasibility of devising a static approach to flaky test prediction. Hence, in this paper, first we extend our preliminary work by replicating the initial analyses on the FlakeFlagger dataset,^2^ in an effort of increasing the generalizability of our results. Secondly, we devise a static flaky test prediction model that can identify flaky tests only considering the design of test cases. Last but not least, we conduct an empirical study that analyzes the performance of the devised model, other than comparing it with two baseline approaches based on source code vocabulary and a mixture of static and dynamic analysis. The key findings of the paper show that static features can be used to characterize flaky tests: this is especially true for metrics and smells connected to source code complexity. In addition, the newly devised machine learning model achieves performance up to 74% in terms of F-Measure, being no worse than techniques that adopt more complex and/or dynamic computations. Perhaps more importantly, our approach is, overall, more precise than the others, therefore minimizing the risks of developers wasting time in diagnosing wrong recommendations. As such, we conclude that the proposed model can represent a more practical solution, which makes the flaky test prediction problem more scalable. To sum up, our work provides the following novel contributions:

1. We provide a large-scale empirical investigation of the distribution of static features in flaky and non-flaky tests, showing their individual and combined effects on the likelihood of a test to exhibit a flaky behavior;
2. We devise the first fully static machine learning approach for flaky test prediction, which relies on the design of test cases and ensures performance comparable with other, more sophisticated techniques previously proposed;
3. We release a publicly available replication package (Pontillo et al. 2022), where we provide access to data, scripts, and results of our experiment. These data can be used by other researchers to verify, replicate, and further investigate the relation between static features and flaky tests.

### Structure of the paper

Section 2 overviews the background and the related literature, summarizing how our work differs from the previous ones. Section 3 describes the research questions and the context, while Section 4 reports on the empirical variables of the study. Sections 5 to 8 describe the methodology and the results that address our research questions, while Section 9 describes the threats to validity of our study, other than the mitigation strategies applied. Section 10 concludes the paper and outlines our future research agenda.

## Background and related work

This section describes the background and the related work that are the foundations of our contributions.

### Terminology

We provide in the following the definitions of the main elements and concepts targeted by our empirical investigation. In particular:

**‘Test case’.**: A test case is defined as *“a set of inputs, execution conditions, a pass/fail criterion, an execution environment, its dependencies, and the corresponding production code”*. This is an extended version of the 829-1998 IEEE standard definition of test case (Association IS 1998): according to previous work (Eck et al. 2019; Luo et al. 2014), the definition includes the additional factors that may play a role in the specific context of test code flakiness, like execution environment, test dependencies, and corresponding production code.
**‘Regression testing’.**: Regression testing is defined as *“the verification activity that allows developers to control newly committed code for the presence of defects”* (Wong et al. 1997). Our work focuses on regression testing activities, as the datasets employed were originally collected by means of multiple re-runs of test cases against the change history of the considered projects (more details later in Section 3.2). The granularity of our experiments is at *unit* test code level, which means that we target test cases that aim at exercising individual components of the production code (Pezze and Young 2008).
**‘Flaky test’.**: A flaky test is defined as *“a non-deterministic test that exhibits both a passing and failing behavior when run against the same code*. We followed the definition provided by Luo et al. (Luo et al. 2014), who also indicated that test code flakiness may arise because of multiple root causes pertaining to how the test code is designed, executed, or dependent from other code.

### Related work

The problem of flaky tests is becoming more and more serious for both researchers and developers (Fowler 2011; Micco 2017). Harman and O’Hearn (Harman and O’Hearn 2018) even suggested that *all* tests should be considered flaky, recommending the development of tools and techniques that can automatically assess the likelihood of a new test becoming flaky in the future. Comprehensive analyses of the state of the art were presented in the recent systematic literature reviews conducted by Parry et al. (Parry et al. 2021) and Zheng et al. (Zheng et al. 2021).

A first research angle frequently treated concerns with the identification of the root causes making tests flaky. In this respect, Luo et al. (Luo et al. 2014) manually inspected 1,129 commits to elicit a taxonomy reporting ten root causes of test flakiness. Thorve et al. (Thorve et al. 2018) conducted a similar study in the context of Android apps, concluding that some root causes are similar to those identified by Luo et al. (Luo et al. 2014), while others relate to program logic and UI. Eck et al. (Eck et al. 2019) built upon these papers to identify additional root causes, shedding light on the potential contribution provided by production code factors. When defining the independent variables to consider in our study, we took the work by Eck et al. (Eck et al. 2019) into account and computed a number of production code metrics and smells. Furthermore, the relation between design issues in test cases, a.k.a. test smells (van Deursen et al. 2001), and test flakiness was observed by Camara et al. (2021a). As explained later in the paper, this was the main reason why we also considered test smells as independent variables of the study. Still on the empirical side, Gruber et al. (Gruber et al. 2021) proposed a new dataset of 7,571 flaky tests in Python, which were identified by rerunning the test suites 400 times; the authors also suggested that flakiness is equally prevalent in Python as it is in Java.

Among the various causes of flakiness, the order dependency one has gained more attention. While Zhang et al. (Zhang et al. 2014) proposed an empirical study on the test independence assumptions, several techniques have been proposed to detect these types of flaky tests: for instance, Gyori et al. (Gyori et al. 2015) proposed a technique for finding shared states between tests, while Bell et al. (Bell et al. 2015) proposed an approach to detect all dependencies between test cases in large projects. More recently, Shi et al. (Shi et al. 2019) proposed iFixFlakies, a tool that automatically fixes real order-dependent tests. The authors evaluated this tool on 58 flaky tests and the tool has correctly fixed all of them. With respect to the research on test order dependency, it is worth clarifying that the goal of the approach proposed in our experimentation is that of predicting the emergence of a flakiness behavior, rather than focusing on the classification of the root cause leading to flakiness. As such, even though issues concerned with test order dependency might be potentially predicted by means of our approach, it cannot report whether a problem identified is actually due to this root cause.

Interestingly, researchers and practitioners have been also working together on the investigation of flaky tests. There is indeed a growing number of industrial studies that propose empirical investigations and tools. Lampel et al. (Lampel et al. 2021) proposed a new approach that automatically classifies failing jobs as pertaining to software bugs or flaky tests. Rehman et al. (Rehman and Rigby 2021) quantified how often a test fails without finding any defect in production code by means of an empirical investigation across four large projects at Ericsson.

In this practitioner’s context, there is also a growing number of studies that target the developer’s opinion. Habchi et al. (Habchi et al. 2021) conducted an interview study involving 14 industrial practitioners. Their results confirmed the problem’s relevance, but also pointed out that in a non-negligible amount of times, flakiness stems from interactions between the system components, the testing infrastructure, and other external factors. Still, on a similar line of research, Gruber and Fraser (Gruber and Fraser 2022) surveyed 335 professional software developers and testers in different domains; their results confirmed the relevance of the problem especially using automated testing.

Another relevant research area pertains to the proposal of tools and techniques to automatically detect flaky tests. Bell et al. (Bell et al. 2018) proposed DeFlaker, a tool that analyzes the differences in code coverage between one commit and another to alert developers of the emergence of some sort of flakiness. Lam et al. (Lam et al. 2019) introduced iDFlakies, a tool that detects flaky tests by rerunning tests in different orders. It is important to note that, besides proposing novel techniques, these studies also publicly released datasets that represented the starting point of later research.

By design, DeFlaker and iDFlakies are able to detect flakiness only after its emergence, namely only after that the developers have introduced flaky tests. In this sense, they could be useful to diagnose flaky tests, but not for preventing their introduction. For this reason, a recent trend concerns the definition of predictive methods that could alert developers of the possible introduction of test flakiness in advance by looking at the static and/or dynamic characteristics of tests. FlakeFlagger (Alshammari et al. 2021) considered static and dynamic features to predict flakiness. In this work, Alshammari et al. (Alshammari et al. 2021) also released their dataset, which was built by executing the same tests 10,000 times and identifying possible non-deterministic behaviors. Bertolino et al. (Bertolino et al. 2021), Pinto et al. (Pinto et al. 2020) and their replications (Camara et al. 2021b; Haben et al. 2021) worked on an orthogonal approach, proposing approaches based on the vocabulary contained in a test method body. They only relied on textual metrics, without considering other features.

With respect to the studies discussed above and the results obtained from our previous feasibility study (Pontillo et al. 2021), our work can be considered as complementary, since it contributes with an additional technique to predict test flakiness that only considers static metrics. It is important to emphasize that our research is driven by a key consideration: a prediction only based on static metrics could lead to benefits in terms of (1) computational costs, as it would avoid the computation of dynamic metrics that would require the execution of the entire test suite; (2) interpretability, as it would allow developers to focus on a refactoring of test cases guided by the static metrics and smells that impact more the likelihood of the test becoming flaky.

## Research questions and context selection

The *goal* of the study was to investigate to what extent a fully static approach can predict the presence of flaky tests, with the *purpose* of assisting developers in the scalable identification of test flakiness. The *perspective* is of both researchers and practitioners: the former are interested in understanding the capabilities of a prediction model based on code- and test-related static metrics when it comes to the identification of flaky tests; the latter are interested in evaluating which are the features more connected to flakiness and that, therefore, should be kept under control when evolving source code.

### Research questions

Our study was structured around four research questions. We started by considering both test and production code metrics and smells. Some of these metrics were related to the size and complexity of both production and test code, e.g., *McCabe cyclomatic complexity* or the number of lines of the test suite (*TLOC*). Other metrics pertained to bad programming practices applied while developing either production or test code. For instance, we considered production code smells (Fowler 2018) such as *Complex Class* and *Spaghetti Code*, other than test smells (van Deursen et al. 2001) like *Eager Test* and *Resource Optimism*.

While the research community has identified test-related aspects as those primarily connected to the potential flakiness of test code (Luo et al. 2014), we considered production code metrics based on the findings reported in a recent work by Eck et al. (Eck et al. 2019). We chose this dimension because in a non-negligible number of cases, the root-cause of test flakiness might be due to errors done in the production code, e.g., when managing concurrency. This reasoning let us define our **RQ**_**1**_: we started by analyzing how the above mentioned metrics correlate to test flakiness. We focused on their individual effect by statistically comparing how their values differ in the sets of flaky and non-flaky tests. We asked: 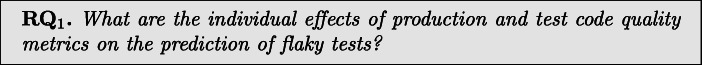


While the results of the first research question might already provide insights into the relations between static metrics and test flakiness, we performed an additional step with the aim of verifying whether the differences observed in **RQ**_**1**_ were still statistically significant when the considered metrics were combined: as shown in literature (Pecorelli et al. 2021), this step is required to establish unbiased conclusions on the capabilities of metrics for predictive models: 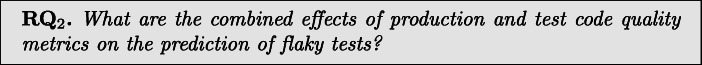


Afterwards, we went beyond the statistical analyses and verified the actual effectiveness of static metrics for the prediction of flaky tests. This led to the definition of a fully static solution that can identify flaky tests, hence allowing us to measure how good static metrics are at predicting flakiness. We then evaluated the performance of the proposed approach. Hence, we asked our third research question: 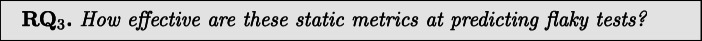


As a final step of our empirical investigation, we compared the prediction performance of the proposed static approach to existing techniques, in an effort of understanding how close are the capabilities of an approach only based on the design of test cases with respect to approaches that employ more seemingly accurate dynamic or textual metrics. The last research question therefore assessed the extent to which our approach may be feasible in practice, namely whether it can be useful in comparison to other existing approaches. Indeed, should other approaches perform notably better than ours, this would imply that a practitioner should not prefer our solution but rather go for alternative approaches. Hence, we asked our final research question (**RQ**_**4**_): 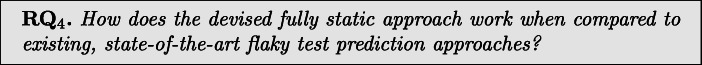


The outcome of our research aimed at enlarging the current body of knowledge on flaky test prediction, providing insights into the value of design-related characteristics for the detection of test flakiness, other than a quantitative assessment of static flaky test prediction with respect to existing techniques. In terms of reporting, we followed the *ACM/SIGSOFT Empirical Standards*^3^ and, in particular, the *“General Standard”* and *“Data Science”* guidelines.

### Context of the study

The *context* of our study consisted of Java open-source projects that belong to the iDFlakies and FlakeFlagger datasets.

The rationale behind the selection of these datasets was driven by their availability, other than their diversity. In particular, the projects are all available on GitHub and are developed by different communities—seven projects belong to the Apache Software Foundation. Furthermore, the projects have a size ranging from some hundreds to one million lines of code. In particular, we analyzed 24 projects coming from FlakeFlagger dataset and 82 projects deriving from iDFlakies dataset. Seven of these projects were in common between the two datasets, yet they referred to different commits: for this reason, we did not have duplicates and, therefore, took all projects into account. Looking at the scope of the various projects, we observed that they vary very much, e.g, some projects relate to http requests and responses, other to container orchestration. A full report of the domains of the considered projects is available in our online appendix (Pontillo et al. 2022). Nonetheless, the domain observations were already insightful to understand that test code flakiness is a widespread problem that affects projects independently from the domain. In terms of testing activities, all the projects make use of a continuous integration pipeline that allows code changes to be verified against a test suite. With the use of a build tool, e.g., Maven, developers can configure the test cases that must be run when new changes are pushed onto the repository. While we cannot know whether the developers of the considered projects defined a test plan document before configuring the tests to run, it is important to notice that all projects establish contribution guidelines that contributors must follow and that include indications on how to conduct testing activities. As such, the testing activities are not left to the developer’s willingness to perform them, but are defined and updated over time. This increases our confidence in the quality assurance procedures adopted by the considered projects.

Perhaps more importantly, we relied on those datasets because of the procedures followed to identify the flakiness information: when populating iDFlakies and FlakeFlagger, Lam et al. (Lam et al. 2019) and Alshammari et al. (Alshammari et al. 2021) indeed ensured the equivalence of test cases and preserved the testing conditions by re-executing test cases in the exact order intended by the developers of those projects. Indeed, they re-run tests following the order and testing conditions established through the build tools.

When addressing the research questions of the study, we considered the two datasets individually, hence reporting the results for each dataset. This was done because the data collection methods used to build the two datasets were different and, therefore, we avoided merging them. In addition, when addressing **RQ**_**4**_, we only focused on the FlakeFlagger dataset since it reported data on the features employed to build the baseline approaches used for comparison (more details are reported in Section 8).

## Empirical study variables

The first step to address the research questions posed in our study concerned with the definition of the empirical study variables, namely (1) the dependent variable to predict and (2) the features to be used as independent variables.

### Dependent variable

The dependent variable of our study is the test flakiness. The information about the flakiness or non-flakiness of a test case is reported in the iDFlakies (Lam et al. 2019) and FlakeFlagger datasets (Alshammari et al. 2021). In particular, test cases are either labeled as *“flaky”* or *“non-flaky”*. As such, our statistical exercise will consider a binary dependent variable.

### Independent variables

The ultimate goal of our work was to verify the extent to which statically computable metrics can be adopted to predict test flakiness. We considered a total of 25 factors along three dimensions i.e., *production and test code metrics*, *code smells*, and *test smells*. Table 1 reports the name and description of the considered metrics, other than the indication of whether they were computed on production or test code. The rationale and motivations for selecting them is discussed in the following.

**Table 1:** List of metrics used as independent variables

Name	Description	Computed on ...
Production and test code metrics		
CBO	Coupling Between Object, i.e., the number of dependencies a class has with other classes (Chidamber and Kemerer 1994).	Production Class
Halstead Length	The total number of operator occurrences and the total number of operand occurrences.	Production Class
Halstead Vocabulary	The total number of distinct operators and operands in a function.	Production Class
Halstead Volume	Proportional to program size, represents the size, in bits, of space necessary for storing the program.	Production Class
LOC	Lines of Code, counting both source and comment lines.	Production Class
LCOM2	Lack of Cohesion of Methods version 2, i.e., the percentage of methods that do not access a specific attribute averaged over all attributes in the class.	Production Class
LCOM5	Lack of Cohesion of Methods version 5, i.e., the density of accesses to attributes by methods.	Production Class
McCabe	It uses to indicate the number of linearly independent paths through a program’s source code (McCabe 1976).	Test Class
MPC	Message Passing Coupling, measures the numbers of messages passing among objects of the class.	Production Class
RFC	Response For a Class, i.e., the number of methods (including inherited ones) that can potentially be called by other classes (Chidamber and Kemerer 1994).	Production Class
TLOC	Number of lines of code of the Test Suite.	Test Class
WMC	Weighted Methods per Class, i.e., the sum of the complexities (i.e., McCabe’s Cyclomatic Complexity) of all the methods in a class (Chidamber and Kemerer 1994). Note that Chidamber and Kemerer (Chidamber and Kemerer 1994) did not define a predefined complexity metric to consider for the computation of WMC. In our case, we opted for the McCabe metric to account for the individual complexity of methods.	Production Class
Code Smells		
Class Data Should Be Private	When a class exposes its attributes, violating the information hiding principle.	Production Class
Complex Class	When a class has a high cyclomatic complexity.	Production Class
Functional Decomposition	When in a class inheritance and polymorphism are poorly used.	Production Class
God Class	When a class has huge dimension and implementing different responsibilities.	Production Class
Spaghetti Code	When a class has no structure and declares long method without parameters.	Production Class
Test Smells		
Assertion Density	Percentage of assertion statements in the test code.	Test Class
Assertion Roulette	When a test method has multiple non-documented assertions.	Test Class
Conditional Test Logic	Conditional code within a test method negatively impacts the ease of comprehension by developers.	Test Class
Eager Test	When a test method invokes several methods of the production object.	Test Class
Fire and Forget	A test that is at risk of exiting prematurely because it does not properly wait for the results of external calls.	Test Class
Mystery Guest	When a test method utilizes external resources (e.g., files, database, etc.).	Test Class
Resource Optimism	When a test method makes an optimistic assumption that the external resource (e.g., File), utilized by the test method, exists.	Test Class
Sensitive Equal.	When the toString method is used within a test method.	Test Class

#### Production and test code metrics

This set is composed of ten factors measuring the size and complexity of production and/or test code. Some of these metrics belong to the Object-Oriented metric suite proposed by Chidamber and Kemerer (Chidamber and Kemerer 1994), e.g., coupling between object classes (CBO), while other metrics come from other catalogs, e.g., the McCabe cyclomatic complexity (McCabe 1976) or the Halstead’s metrics (Murillo-Morera and Jenkins 2015). The rationale behind the selection of these metrics was driven by our willingness to verify whether large and/or complex code might have an impact on the likelihood of observing a flaky behavior of the test case. In addition, previous analyses (Pecorelli et al. 2021, 2022) investigated those metrics to understand the robustness of test code. In this sense, our study can complement previous findings through an understanding of the role of production and test code metrics for test flakiness. More particularly, we computed *TLOC* and *McCabe* on the test code, while the other eight metrics were computed on the production code. To compute these metrics, we relied on a tool that we have developed within our research lab and that was used for a number of previous studies (e.g., Pecorelli et al. 2019, 2021, 2022). Its use was not only motivated by our familiarity with the instrument, but also because of the extensive testing activities we could perform on this tool over the years. For the sake of replicability, we made this tool available in our online appendix (Pontillo et al. 2022).

#### Code smells

These indicate the presence of sub-optimal solutions to the development of source code (Fowler 2018) that might contribute to the increase of technical debt (Palomba et al. 2018). It is reasonable to believe that writing tests for smelly code may be harder and might possibly lead them to be less effective—this was somehow showed by Grano et al. (Grano et al. 2019). Hence, we run our own instance of Decor (Moha et al. 2009), a state-of-the-art code smell detector, to count the number of instances of five code smell types having different characteristics and targeting well-known design issues, i.e., *Class Data Should Be Private*, *Complex Class*, *Functional Decomposition*, *God Class*, and *Spaghetti Code* (Fowler 2018). Our tool implements the original rules proposed by Moha et al. (Moha et al. 2009). These code smells were computed on production code only, as our goal was to consider the potential effect that design issues in production code have on the likelihood of tests to be flaky. While other code smell detectors have been proposed in literature (Azeem et al. 2019; de Paulo Sobrinho et al. 2018), we opted for Decor for three main reasons. First, it has been widely experimented in literature, showing good detection performance (Moha et al. 2009; Palomba et al. 2014; Palomba et al. 2017). Secondly, it might be employed when performing large-scale studies, given its lightweight nature (Tufano et al. 2017). Third, its usage allowed us to focus on a larger variety of code smell types: other detectors can indeed identify a lower amount of code smells (dos Reis et al. 2021). To enable replications, we made our own version of Decor accessible in our online appendix (Pontillo et al. 2022).

#### Test smells

Similarly to code smells, these are defined as bad programming practices in unit test code (van Deursen et al. 2001). As originally defined, test smells may indeed reveal the presence of issues that induce test flakiness (van Deursen et al. 2001). Moreover, Camara et al. (Camara et al. 2021a) showed a correlation between test smells and flaky tests. For these reasons, we run a state-of-the-art test smell detector named VITRuM (Pecorelli et al. 2020) to verify whether test smells have an impact on flakiness. The detector identifies seven test smell types, i.e., *Assertion Roulette*, *Conditional Test Logic*, *Eager Test*, *Fire and Forget*, *Mystery Guest*, *Resource Optimism*, and *Sensitive Equality*. Also in this case, it is worth pointing out that other detectors have been proposed over the last decade (Greiler et al. 2013; Lambiase et al. 2020; Palomba et al. 2018; Peruma et al. 2020; Van Rompaey et al. 2007). In this case, the selection of VITRuM was driven by two observations. On the one hand, this is a tool we have a direct access to and, for this reason, we could directly interact and run it against the considered datasets. On the other hand, the tool implements multiple test smell types that have been originally associated to test flakiness, hence allowing us to assess their actual relation to flaky tests.

When computing metrics and smells on production code, we had to link test cases to their correspondent production code—otherwise, we could not investigate the value of the production code metrics. In this respect, we used a pattern matching approach based on naming conventions and already used in previous work (e.g., (Grano et al. 2019; Haben et al. 2021; Pecorelli et al. 2021)). This approach simply uses the name of a production class (e.g., *‘ClassName’*) as the base for finding the corresponding test class, which will be the one whose name is the same as the one of the production class, but includes the prefix/postfix *‘Test’* (e.g., *‘TestClassName’* or *‘ClassNameTest’*). Whenever this pattern matching failed, the production class associated with the test class could not be detected and, for this reason, we had to discard the test from our analysis. Despite this practical limitation, the selection of this pattern matching approach was mainly driven by the good compromise between accuracy and scalability that it ensures; more complex approaches, e.g., those based on static and dynamic slicing (Qusef et al. 2013), can be hardly employed on a large scale. In an effort of conducting a larger experimentation of our approach, we therefore accepted the intrinsic limitation of the pattern matching method and excluded the tests/projects where the developers did not use the appropriate naming conventions.

The outcome of this linking process led to the modification of the initial datasets. In particular, we had to discard five projects from the FlakeFlagger dataset and 31 projects from the iDFlakies one. In all these cases, the developers did not follow the above-mentioned naming conventions, hence not allowing us to properly link production and test classes. As for the remaining projects, the outcome of the linking process led us to the removal of some test cases, including all methods called *‘setUp’* and *‘tearDown’*—these represent fixtures that only enable the correct allocation an de-allocation of the resources to be used by the tests and could not clearly linked to any production class. As a consequence of these filtering actions, the iDFlakies dataset finally contained 44,592 test cases (including 281 flaky tests) pertaining to 51 projects, while the FlakeFlagger dataset contained 10,914 test cases (including 671 flaky tests) of 19 projects. For the sake of verifiability, in our online appendix (Pontillo et al. 2022) we reported the list of the projects discarded from the analysis.

## RQ_1_ - The individual effects of metrics on test flakiness

This section discusses the research methodology and the results achieved when we analyzed the individual effects of metrics considered.

### Research methodology

We assessed if the independent variables were different in the set of flaky and non-flaky sets in both datasets. As a first step, we normalized the metric values through the *min-max scaling*—this was needed because the metric values came in different range of values and, as such, we used a min-max scaling to have them under the same representation range (Han et al. 2011). We reported in our *online appendix* (Pontillo et al. 2022) the updated dataset used to address the research question.

We showed boxplots depicting the distribution of the metrics and smells. Then, we computed the Mann-Whitney and Cliff’s Delta tests to verify the statistical significance of the observed differences and their effect size. The choice of non-parametric methods came from the verification of the normality of the distributions. The data indeed followed a non-normal distribution even after the min-max scaling normalization.

**Fig. 1:**
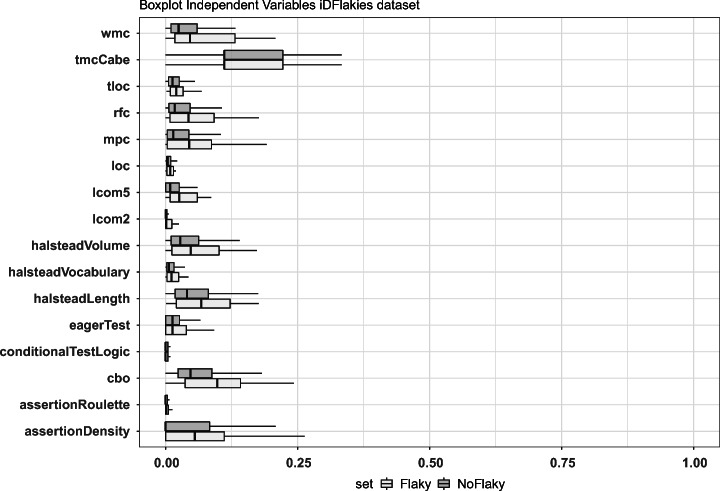
**RQ**_**1**_. Analysis of the metric profiles of flaky and non-flaky tests on the iDFlakies dataset

**Fig. 2:**
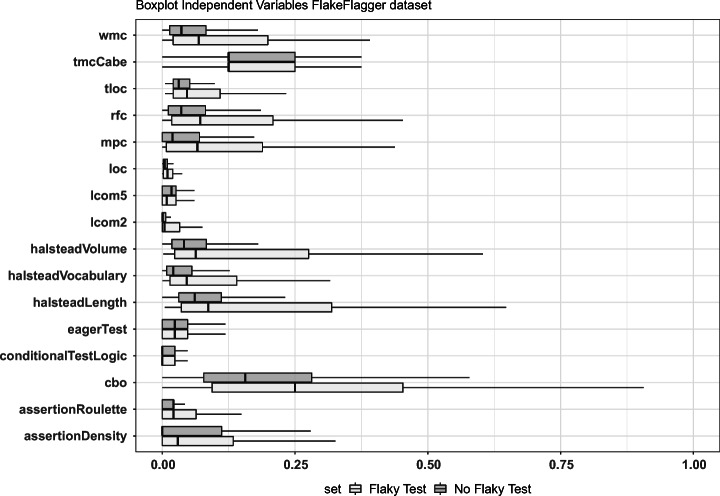
**RQ**_**1**_. Analysis of the metric profiles of flaky and non-flaky tests on the FlakeFlagger dataset

### Analysis of the results

Figures 1 and 2 depict the boxplots of the distributions of metrics and smells which exhibit some differences between the sets of flaky and non-flaky tests in the two datasets. The boxplots showing all factors are reported in our replication package (Pontillo et al. 2022). We can observe that some factors vary in the two sets in both boxplots: this is especially true when considering the production and test code metrics for which the medians of flaky tests and corresponding production code are often higher than those of non-flaky tests. These results confirm that flaky tests have a different metric profile than other tests. In particular, we observe differences in terms of control flow graph-related metrics (e.g., production WMC metric computed on tests) and complexity of the expressions used in the code (e.g., the Halsteald’s metrics). This seems to suggest that the development of test cases is heavily impacted by complexity measures, possibly increasing the likelihood to induce flakiness. As for the test-related factors, the higher median of assertion density in the flaky test set might be connected to the fact that having more assertions increases the chances to induce flakiness due to restrictive ranges in the values compared within assert statements (Eck et al. 2019). Finally, we observe the severity of the *Eager Test* smell as a metric that differs in two sets as distribution but not as median. This smell measures how focused a test is, namely whether it exercises more methods of the production code. Based on our results, we may conjecture that the lack of focus of tests does not allow them to properly set the environment needed to exercise the production code: as a consequence, their outcome may depend on the order of execution of test methods, i.e., the outcome may change if the environment is (not) set before calling the smelly test.

The results of the statistical tests are reported in Tables 2 and 3 and confirm the discussion provided so far. Most of the metrics (17 for the first dataset, 22 for the second) presented a *ρ*-value< 0.05, meaning that the differences between the distributions of flaky and non-flaky tests are statically significant. These differences have, however, a small effect size in 14 cases for the first dataset and in 12 cases for the second dataset. When combining the boxplots with the statistical results, we could observe cases where the distributions were very similar yet statistically different, possibly indicating interpretation errors. These are, for instance, the cases of the McCabe metric and the *Conditional Test Logic* smell. We took a closer look at these cases, finding that the differences among the distributions were so small that they could not be visible with a boxplot representation. Nonetheless, some statistical differences still arose. As an example, the Cliff’s Delta test for *Conditional Test Logic* reported negligible differences, while the test for the McCabe metric reported small differences. This analysis reinforced the need for considering both boxplots and statistical perspectives to better interpret our findings.

**Table 2:** Mann-Whitney and Cliff’s Delta Tests for the iDFlakies dataset. N, S, M, and L indicate negligible, small, medium and large effect size, respectively. Significant p-value and *δ* value are reported in bold-face

Statistic tests					
	p-value	*δ*		p-value	*δ*
CBO	***1.34*e**^−***13***^	**S**	Complex Class	**9.85**^−**11**^	N
Halstead Length	***1.17*e**^−**06**^	**S**	FD	**0.03**	N
Halstead Vocab.	***4.70*e**^−**09**^	**S**	God Class	0.38	N
Halstead Volume	***3.78*e**^−**07**^	**S**	Spaghetti Code	***8.47*e**^−**11**^	N
LOC	***7.84*e**^−**11**^	**S**	Assertion Density	***1.69*e**^−**8**^	**S**
LCOM2	< **2.2e**^−**16**^	**S**	Assertion Roulette	***3.81*e**^−**10**^	**S**
LCOM5	***1.63*e**^−**14**^	**S**	Cond. Test Logic	0.10	N
McCabe	0.20	N	Eager Test	***2.03*e**^−**13**^	**S**
MPC	***1.04*e**^−**7**^	**S**	Fire and forget	0.74	N
RFC	***6.56*e**^−**11**^	**S**	Mystery Guest	0.40	N
TLOC	***1.16*e**^−**8**^	**S**	Resource optimism	0.12	N
WMC	***1.80*e**^−**12**^	**S**	Sensitive equality	0.17	N
CDSBP	***1.30*e**^−**9**^	N

**Table 3:** Mann-Whitney and Cliff’s delta tests for the FlakeFlagger dataset. N, S, M, and L indicate negligible, small, medium and large effect size, respectively. Significant p-value and *δ* value are reported in bold-face

Statistic tests					
	p-value	*δ*		p-value	*δ*
CBO	< ***2.2*e**^−***16***^	**S**	Complex Class	< ***2.2e***^−***16***^	N
Halstead Length	< ***2.2*e**^−***16***^	**S**	FD	0.049	N
Halstead Vocab.	< ***2.2*e**^−***16***^	**S**	God Class	***7.7*e**^−***4***^	N
Halstead Volume	< ***2.2*e**^−***16***^	**S**	Spaghetti Code	< ***2.2*e**^−***16***^	N
LOC	< ***2.2*e**^−***16***^	**S**	Assertion Density	***5.09*e**^−***4***^	N
LCOM2	< ***2.2*e**^−***16***^	**S**	Assertion Roulette	***4.28*e**^−***3***^	N
LCOM5	< ***2.2*e**^−***16***^	N	Cond. Test Logic	***3.91*e**^−***7***^	N
McCabe	< ***2.2*e**^−***16***^	**S**	Eager Test	0.93	N
MPC	< ***2.2*e**^−***16***^	**S**	Fire And Forget	***8.73*e**^−***14***^	N
RFC	< ***2.2*e**^−***16***^	**S**	Mystery Guest	< ***2.2*e**^−***16***^	**S**
TLOC	< ***2.2*e**^−***16***^	**S**	Resource Optimism	0.10	N
WMC	< ***2.2*e**^−***16***^	**S**	Sensitive Equality	***1.5*e**^−***2***^	N
CDSBP	0.3887	N			

The statistical results are summarized in Table 4 - for each metric, a gray cell represents that it is statistically significant on a dataset; white otherwise. Looking at the table, we can observe that there are some differences between the two datasets, some metrics are statistically significant only in iDFlakies dataset, i.e., *Class Data Should Be Private,* and *Eager Test*, while other metrics are statistically significant only in FlakeFlagger dataset, i.e., *McCabe, God Class, Conditional Test Logic, Fire and Forget, Mystery Guest,* and *Sensitive Equality*. These differences may depend on the different nature of the datasets, e.g., the number of flaky tests or the number of test cases, yet there are still a number of metrics that are statistically significant in both datasets, such as those related to code complexity.

**Table 4:**
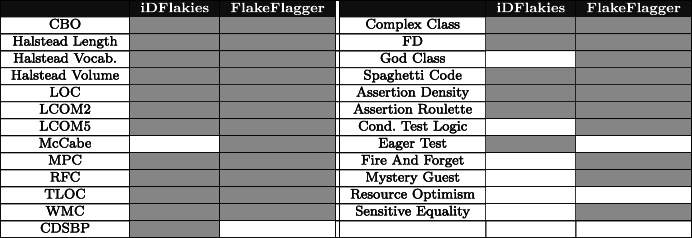
Summary of statistical significance of metrics between the two datasets. The gray color indicates that a metric is statistically significant in the dataset, while it is white otherwise

Finally, we identified the presence of *Assertion Roulette* smell instances to be statistically significant in both datasets, while other smells are often significant in only one of them. 
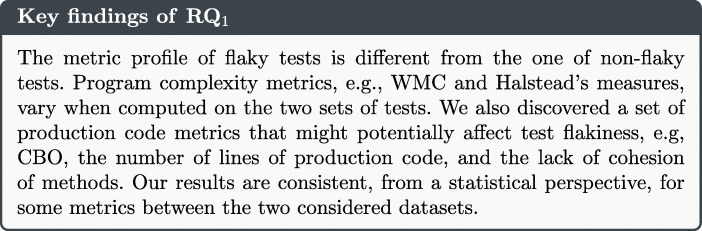


## RQ_2_ - The combined effects of metrics on test flakiness

This section discusses the research methodology and the results achieved when we analyzed the combined effects of metrics considered.

### Research methodology

After studying the statistical significance of the distributions of our independent variables in both datasets, we proceeded with our second research question. In particular, **RQ**_***2***_ aimed at assessing whether the statistically significant factors identified in the previous research question were still significant when combining all metrics: this analysis was required since the individual effect of a factor might be reduced (or even lost) when other factors come into play (Pecorelli et al. 2021). Hence, we took the normalized datasets into account and devised a *Logistic Regression Model*, which belongs to the class of *Generalized Linear Model* (GLM) (Nelder and Wedderburn 1972). We have used this statistical modeling approach because it does not assume the distribution of data to be normal. In fact, we verified the normality of the distribution by means of the K-S Lilliefors test (Garson 2012), which failed to reject the null-hypothesis, i.e., our data is not normally distributed. Furthermore, the *Logistic Regression Model* can deal with dichotomous dependent variables, hence fitting our case.

More formally, let *Logit*(*π*_*f*_) be the explained test flakiness *f*, let *β*_0_ be the log odds of the likelihood of flakiness being increased in a test, and let the parameters *β*_1_ ⋅ *f*_1_, *β*_2_ ⋅ *f*_2_, …, *β*_*n*_ ⋅ *f*_*n*_ be the differentials in the log odds of being the likelihood of flakiness increased for a test with characteristics *f*_1_, *f*_2_, …, *f*_*n*_, the statistical model is represented by the function:

1 $$ Logit(\pi_{f}) = \beta_{0} + \beta_{1} \cdot f_{1} + \beta_{2} \cdot f_{2} + {\dots} + \beta_{n} \cdot f_{n}. $$

To implement the model, we relied on the glm function available in R toolkit.^4^ Moreover, to avoid multi-collinearity we used the vif (Variance Inflation Factors) function implemented in R to discard highly correlated variables, putting a threshold value equal to 5 (O’brien 2007). The interested reader can find additional information on the correlation between the independent variables in our online appendix (Pontillo et al. 2022). In particular, we conducted correlation analyses using the non-parametric Spearman’s rank correlation coefficient (Myers and Sirois 2004) with the aim of providing further insights into the relations between the considered variables. As a result, we found out that such a correlation analysis reinforced the results obtained when using the vif function, hence making us more confident about the decisions made when discarding variables.

### Analysis of the results

Table 5 reports the results of the *Logistic Regression Model* on the iDFlakies dataset. As the reader might observe, Table 5 reports only 17 of the independent variables; the other eight factors, i.e., Halstead Length, Halstead Volume, LCOM2, LOC, MPC, RFC, WMC, and Spaghetti Code, were excluded by the model as a result of the vif analysis. Similarly, Table 6 reports the results of the *Logistic Regression Model* on the FlakeFlagger dataset, in which are shown only 16 of the independent variables; the other nine factors, i.e., Complex Class, Halstead Length, Halstead Volume, LCOM2, LOC, MPC, RFC, WMC, and Spaghetti Code, were excluded as a consequence of the multi-collinearity checks.

**Table 5:** Results for **RQ**_***2***_ achieved by the statistical model and obtained with iDFlakies dataset

Generalized linear model							
	Estimate	S.E.	Sig.		Estimate	S.E.	Sig.
Intercept	-4.06	2.09	.	Cond. Test Logic	-44.82	13.15	***
TLOC	6.59	2.34	**	Fire and Forget	0.88	1.98	
McCabe	1.06	0.67		LCOM5	-1.71	1.15	
Assertion Density	1.41	0.57	*	CBO	0.34	0.77	
Assertion Roulette	-23.64	9.03	**	Halstead Voc.	3.69	0.97	***
Mystery Guest	-1.04	2.69		CDSBP	1.99	1.71	
Eager Test	4.91	0.97	***	Complex Class	1.11	0.63	.
Sensitive Equality	-7.42	7.53		FD	-0.57	0.41	
Resource Optimism	-4.18	4.51		God Class	-1196.50	1867.19

**Table 6:** Results for **RQ**_***2***_ achieved by the statistical model and obtained with FlakeFlagger dataset

Generalized linear model							
	Estimate	S.E.	Sig.		Estimate	S.E.	Sig.
Intercept	-11.63	168.77		Cond. Test Logic	-2.22	1.14	.
TLOC	4.95	0.78	***	Fire and Forget	3.10	0.97	**
McCabe	2.58	0.40	***	LCOM5	-19.08	2.78	***
Assertion Density	0.53	0.44		CBO	0.61	0.26	*
Assertion Roulette	0.29	0.85		Halstead Voc.	5.58	0.57	***
Mystery Guest	6.55	0.55	***	CDSBP	-1.74	0.84	*
Eager Test	-7.16	1.12	***	FD	-0.16	0.20	
Sensitive Equality	-1.13	1.13		God Class	176.33	3657.57	
Resource Optimism	-6.63	1.42	***				

For each variable, the tables report the value of the estimate, the standard error, and the statistical significance. The latter is explained by the number of stars, i.e., ‘⋆⋆⋆’ indicates a *p*< 0.001, ‘⋆⋆’ indicates a *p*< 0.01, ‘⋆’ indicates a *p*< 0.05, and ‘.’ indicates a *p*< 0.1.

For the sake of understandability, we split the following discussion according to the categories of metrics analyzed.

#### Results for production and test code metrics

Looking at Table 5, only one metric, namely the test lines of code (TLOC), was statistically significant on the iDFlakies dataset. The value of the estimate was positive (6.56), meaning that an increase of lines of test code statistically leads to an increase of the likelihood of the test being flaky. TLOC was a relevant metric in the context of the FlakeFlagger dataset too (Table 6), hence confirming that longer tests are statistically associated to test flakiness. Besides the lines of test code, we could observe other statistically significant factors on this dataset. These pertain to various aspects of production code quality, like cohesion, coupling, and complexity. The LCOM5 estimate was equal to -19.08: the negative estimate of the metric indicates that an increase in LCOM5 values corresponds to a decrease of the likelihood of tests being flaky. In turn, higher LCOM5 values indicate lower cohesion, i.e., the LCOM5 is an inverse metric. Hence, we can conclude that the lower the cohesion the lower the likelihood of tests being flaky. This result looks unexpected and points out the need for further analyses of how cohesion influences software testability. On the other side, coupling (CBO) and complexity metrics (McCabe and Halstead Vocabulary) had a positive correlation to flaky tests. Also, in this case, the results seem to highlight the relevance of production code maintainability for source testability: an increase in coupling and complexity may indeed make harder for developers to verify the source code, potentially leading to the introduction of flakiness.

#### Results for code smells

When analyzing the correlation between code smells and flakiness, we could delineate a limited relation. Both Tables 5 and 6 show that most of the code smells were not statistically significant. Particularly interesting was the case of *God Class* (also known as *Blob*), which appears when a class is poorly cohesive and maintainable (Fowler 2018): because of its properties, the code smell has been often associated to various forms of technical debt (Khomh et al. 2012; Palomba et al. 2018), including a decrease of the overall effectiveness of test cases (Grano et al. 2019; Spadini et al. 2018). According to our results, the negative effects of *God Class* do not increase the likelihood of the corresponding tests being flaky. The only two exceptions to this general discussion were *Complex Class* on the iDFlakies dataset and *Class Data Should be Private* on the FlakeFlagger dataset. While the presence of a high cyclomatic complexity seems to confirm the results obtained in **RQ**_***1***_, the second does not have obvious connections to flakiness. Looking at the definition, this smell affects classes that do not encapsulate fields, hence providing public access to their attributes. To provide an interpretation of this finding, we manually dived into the Flake Flagger dataset and analyzed a sample of the production classes affected by this smell. We randomly selected 20 classes affected by each smell and tried to establish a motivation for the statistical results obtained—this process was mainly conducted by the first author of the paper, who was supported by the other authors whenever needed. As a result, we could discover that the examined classes had, however, high cyclomatic complexity and, most likely, the statistical significance was due to a *casual reflection* of the high co-occurring complexity. In other words, it is not the presence of this code smell to directly influence the test flakiness but rather a co-occurring phenomenon. We believe this is reasonable, as code smell capture orthogonal dimensions with respect to complexity metrics.

#### Result for test smells

We observed different - or even contrasting - results when considering test smells over the two considered datasets. The first discussion concerns *Eager Test*, which appeared to be positively correlated (estimate= 5.07) on the iDFlakies dataset and negative correlated (estimate=-7.16) with test flakiness on the FlakeFlagger one. This smell arises when a unit test exercises more production methods, hence not being focused on a specific target (van Deursen et al. 2001), and has been previously correlated to a decrease of test code effectiveness (Spadini et al. 2018). Our findings are not definitive, as flakiness appears to be impacted by the lack of focus of the *Eager Test* smell depending on the cases. In this sense, it is reasonable to believe that co-occurring phenomena might affect the likelihood of tests to be both smelly and flaky. Further empirical investigations might therefore analyze these phenomena further.

Test smells such as *Conditional Test Logic* and *Assertion Roulette* were negatively correlated to flakiness on the iDFlakies dataset, meaning that an increasing amount of these smells does not imply an increase of the likelihood of the affected tests to become flaky. On the one hand, the result obtained for *Conditional Test Logic* is somehow unexpected. A test affected by this smell has multiple paths and exercises more execution paths of production code, possibly being more likely to be non-deterministic. Our findings seem to suggest that this is not true in general but, perhaps, only specific circumstances influence the harmfulness of the smell. On the other hand, the presence of an *Assertion Roulette* implies the lack of documentation. Our findings suggest that having multiple non-documented assertions does not risk to become harmful for flakiness. Interestingly enough was, however, the role of the assertion density—which measures the amount of assertions per lines of test code. We found a positive correlation (estimate= 1.43). This indicates that, while missing documentation has a limited connection to flakiness, the presence of too many assertions can potentially impact flakiness.

When analyzing the FlakeFlagger dataset, we found two more positive correlations due to *Fire and Forget* and *Mystery Guest*. The former highlights a technical debt caused by the sub-optimal use of threads: by nature, this smell is related to concurrency and asynchronous wait issues (Camara et al. 2021a), which are among the most diffused root causes of test flakiness (Eck et al. 2019; Luo et al. 2014). The latter refers to the use of external resources within the test code, which make tests more dependent on those resources. Also in this case, the reliance on external sources is known to be a root cause of flakiness (Eck et al. 2019; Luo et al. 2014); our findings suggest that test smell detectors can be a useful means to identify potential cases of flakiness.

In any case, it is worth remarking that the differences noticed between the two statistical models may be attributable to the different size of the datasets, other than to the number of flaky tests present, i.e., 281 in the iDFlakies dataset and 671 in the FlakeFlagger dataset. 
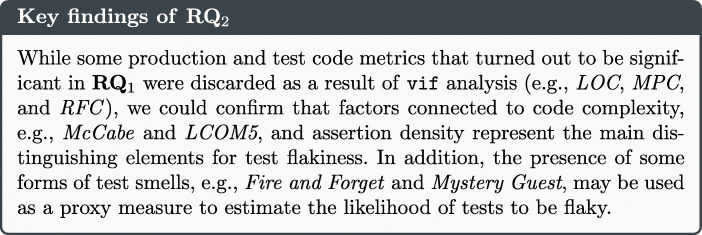


## RQ_3_ - An approach to predict test flakiness statically

While the correlations identified in **RQ**_***2***_ do not necessarily indicate causation, they may suggest some sort of relation between static metrics and test flakiness. The analyses done in **RQ**_***2***_ were indeed preliminary and had the goal to understand whether it is in principle possible to consider static metrics for flakiness prediction. The promising results achieved let us believe that a fully static approach to the prediction of flaky tests would have been possible. Hence, this section discusses the research methodology and the results achieved when exploring such a possibility.

### Research methodology

The methodology employed to address **RQ**_***3***_ concerned with the definition of a machine learning pipeline that would produce reliable measurements of the performance of a static flaky test predictor based on the most relevant metrics explored in our study.

The first step is related to the feature engineering process, that is, the identification of the relevant metrics to use as predictors. While the statistical exercise conducted in the previous research question already provided indications on which features are more connected to test flakiness, it does not necessarily provide insights into the predictive power of the considered metrics (Azhagusundari et al. 2013). In other words, **RQ**_***2***_ only reported correlations, while we were interested in assessing the value of the metrics as features of a machine learner more precisely. Hence, we performed a further step ahead by (1) running the vif analysis to discard highly correlated variables (O’brien 2007); and (2) quantifying the predictive power of each metric in terms of information gain (Quinlan 1986). While the former analysis allowed us to limit the scope of our investigation to the actually relevant features, the latter is a measure of how much a model would benefit from the presence of a certain predictor. More formally, let *P* be the flaky test predictor, let *F* = $\left \{f_{1}, f_{2}, ..., f_{n} \right \}$ be the set of features composing *P*, an information gain algorithm (Quinlan 1986) computes the difference from before to after splitting *P* on an attribute *f*_*i*_ in terms of entropy. It specifically applies the following formula:

2 $$ InfoGain(P, f_{i}) = H(P) - H(P | f_{i}) $$

where the function *H*(*P*) measures the entropy of the model relying on *f*_*i*_ as predictor and the function *H*(*P*|*f*_*i*_) represents the entropy of the model that does not rely on *f*_*i*_ as predictor. The specific measure of entropy is based on the Shannon’s definition (Shannon 1948), namely:

3 $$ H(P) = - \sum\limits_{i=1}^{n} prob(f_{i}) \log_{2} prob(f_{i}) $$

Hence, the algorithm measures how much the uncertainty of the model *P* is reduced because of a predictor *f*_*i*_. In our work, we computed this measure by using the Gain Ratio Feature Evaluation algorithm (Quinlan 1986). This ranks features in descending order of expected information gain, putting the most valuable features at the top. Similarly to previous work in the field (Alshammari et al. 2021; Catolino et al. 2019), we considered the predictors having an information gain higher than zero as those to use for the machine learning exercise, i.e., we discarded the metrics that did not provide any expected beneficial effect on the performance.

Once we had completed the feature engineering process, we proceeded with the identification of the machine learning algorithm to use. The literature on flaky test prediction is still embryonic (Parry et al. 2021) and, for this reason, only a few studies have been conducted on the best classifiers to use. Therefore, we took this as an opportunity to benchmark learning algorithms with different characteristics and making different assumptions on the underlying data. We evaluated Decision Trees (Freund and Mason 1999), Naive Bayes (Webb et al. 2010), Multilayer Perceptron (Taud and Mas 2018), and Support Vector Machine (Noble 2006) as basic classifiers. Additionally, we also considered two ensemble techniques such as Ada Boost (Schapire 2013) and Random Forest (Ho 1995)—the latter was the one used by Alshammari et al. (Alshammari et al. 2021). To implement the algorithms, we employed the Scikit-Learn library (Kramer 2016) in Python, which provides public APIs that let configure, execute, and validate all the above-mentioned classifiers.

In terms of training, we had to deal with the fact that the flaky test problem is an unbalanced problem. The number of flaky test instances represented the 0.9% and 6.8% of the total amount of test cases in the iDFlakies and FlakeFlagger datasets, respectively. As such, the test flakiness was largely underrepresented, threatening the ability of machine learning algorithms to properly learn the characteristics of flaky tests. Hence, we faced the problem by (i) experimenting with multiple under- and over-sampling techniques to balance our data and (ii) comparing them to the results obtained without any balancing technique. As for under-sampling, we made use of NearMiss 1, NearMiss 2, and NearMiss 3 algorithms (Yen and Lee 2006). These techniques first compute the distance between instances of the majority and minority class. Then, they select for removal instances of the majority class that have the shortest distance with instances of the minority class: the underlying idea is indeed that of removing the most similar majority samples to increase the diversity of the training set and, therefore, let a machine learner more appropriately learn features. The three versions of the NearMiss algorithm differ for the distance function used in the first computational step. In addition to these algorithms, we also experimented with a Random Undersampling approach that explored the distribution of majority instances in a random fashion and under-samples them. As for over-sampling, we experimented with *Synthetic Minority Oversampling Technique*, a.k.a. SMOTE (Chawla et al. 2002), and advanced versions of this algorithm such as *Adaptive Synthetic Sampling Approach*, a.k.a. ADASYN (He et al. 2008) and the Borderline-SMOTE (Han et al. 2005). While the basic SMOTE approach uses a simple k-nearest neighbor function to identify the minority class instances to over-sample, ADASYN attempts to over-sample minority class instances according to their level of difficulty in learning. Instead, Borderline-SMOTE builds on top of the concept of borderline examples, namely it selects minority class instances to over-sample based on how similar they are with respect to the instances of the majority class. In addition to these algorithms, we also experimented with a Random Oversampling approach that explores the distribution of minority instances in a random fashion and over-samples them.

We then followed a similar methodology as previous work (Alshammari et al. 2021; Pinto et al. 2020) to evaluate the models. We employed a stratified ten-fold cross validation (Bengio and Grandvalet 2004; Kohavi 1995), applying it on both individual projects and considering all projects as a unique dataset. More particularly, this strategy first randomly partitions the data into ten folds of equal size. Then, it iteratively selects a single fold to use as test set, while the other nine are used as training set. It is important to note that we normalized the metric values through the *min-max scaling* after splitting the training and test sets, namely at each iteration of the ten-fold cross validation - this was required to perform a realistic validation of the model where the training and test sets were individually normalized based on their own distributions. It is worth remarking that we applied the different balancing techniques at each iteration of the cross-validation rather than before evaluating the models. In this way, we could avoid forms of *data leakage* (Shabtai et al. 2012) due to the fact that the resulting test sets would have been balanced, not representing a real-case scenario where the number of flaky tests is way lower than the one of stable tests, i.e., we only balanced the training sets. When training the classifiers, we also optimized the hyper-parameters of the experimented classifiers using the Random Search strategy (Bergstra and Bengio 2012): this is a search-based algorithm that randomly samples the hyper-parameter space in order to find the best combination of hyper-parameters maximizing the F-Measure. For the sake of replicability, we reported the exact hyper-parameter configuration for each classifier in our replication package (Pontillo et al. 2022).

Finally, to evaluate the performance achieved by the experimented models, we relied on three metrics such as precision, recall, and F-Measure. We also statistically verified the validity of our findings exploiting the Nemenyi test (Nemenyi 1963) for statistical significance and report its results by mean on MCM (Multiple Comparison with the best) plots (Koning et al. 2005). As a significance level, we used 0.05; the elements plotted above the gray band in the plots are statistically larger than the others. To perform this last step, we relied on the nemenyi function available in R toolkit.^5^

### Analysis of the results

**Fig. 3:**
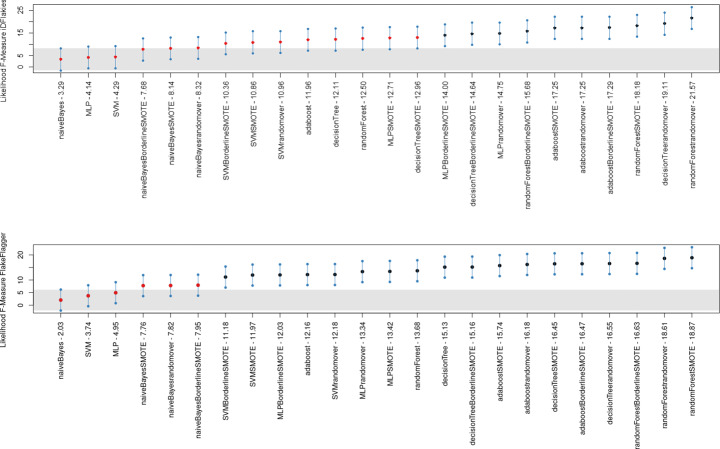
The likelihood of each technique in within prediction in Nemenyi rank in terms of F-Measure. Circle dots are the median likelihood, while the error bars indicate the 95% confidence interval. 60% of likelihood means that a classification technique appears at the top-rank for 60% of the studied projects

We run each machine learning algorithm experimented against the two datasets. For the sake of readability, in this section we mainly focus on the best of those algorithms, while we included the full results in our online appendix (Pontillo et al. 2022). Figure 3 plots the outcome of the Nemenyi test on the two datasets, which were the means we used to decide on the best algorithm to explore further. More particularly, the dots in the figures represent the median F-Measure that the algorithms obtained on the two datasets: a blue dot indicates that the F-Measure of an algorithm is statistically better than the other algorithms, while the red dots indicate that the performances obtained are not statistically different. As shown, for both datasets Random Forest was the best classifier but with a different balancing technique, i.e. Random Oversampling for the iDFlakies dataset and SMOTE for the FlakeFlagger dataset. It is worth remarking that the ADASYN technique does not appear in the figure because it failed on some projects, making the comparison with other techniques unfair. At the same time, the figure does not show the outcome of the models trained with under-sampling methods: these models were all consistently worse than the others and, therefore, we decided not to include them in the figure to ease readability—detailed results are available in our online appendix (Pontillo et al. 2022).

These preliminary results already provide some insights into the capabilities of learning flaky tests. First, we could corroborate previous findings on the highest performance of Random Forest for this problem (Alshammari et al. 2021; Lam et al. 2019). Second, simpler data over-sampling approaches seem to work better than most sophisticated ones. Indeed, Random Oversampling and SMOTE were consistently better on both datasets. The likely reason behind this finding connects to the peculiarities of the data we are considering. Advanced over-sampling techniques are based on the identification of instances which are more difficult to learn (ADASYN) or borderline (Borderline-SMOTE): while future investigations should be conducted on this matter, it is possible that the features characterizing flaky and non-flaky tests are diverse enough not to be considered as hard to learn or borderline, hence making ADASYN and Borderline-SMOTE unable to properly work. Last but not least, it is worth reporting that under-sampling methods always behaved worse than both over-sampling approaches and the no-balance cases. Being the problem of flaky test prediction highly unbalanced, these methods lead to remove way too many samples of the majority class, hence leading to a deterioration of the performance due to the inability to learn neither flaky and non-flaky test characteristics.

Table 7 reports the outcome of the feature engineering process, showing the information gain (IG) obtained when building the Random Forest model. Looking at the two lists, we can observe that for the iDFlakies dataset there are 10 features with an IG> 0.001, while for the Flakeflagger dataset there are 12 features. In addition, the information gain values for the first dataset are lower than those of the second. This might be explained by the nature of the datasets, as iDFlakies contains a lower percentage of flaky tests.

**Table 7:** List of features not excluded by the VIF analysis and with an information gain (IG) higher 0.001 for iDFlakies and FlakeFlagger datasets

iDFlakies dataset		FlakeFlagger dataset	
Features	IG	Features	IG
Halstead Vocabulary	0.0338	Halstead Vocabulary	0.1727
CBO	0.0166	Assertion Density	0.0539
LCOM5	0.0089	CBO	0.0359
Complex Class	0.0059	TLOC	0.0284
Eager Test	0.0059	Mystery Guest	0.0157
TLOC	0.0049	McCabe	0.0133
Class Data Should Be Private	0.0021	LCOM5	0.0128
Assertion Roulette	0.0019	Assertion Roulette	0.0107
Assertion Density	0.0010	Conditional Test Logic	0.0076
McCabe	0.0010	Eager Test	0.0066
		Fire and Forget	0.0013
		Functional Decomposition	0.0011

Analyzing the most relevant features, we could observe that, independently from the dataset, the higher values were related to production and test code complexity measures. This is in line with the results of **RQ**_2_ and confirms that the development of test cases and the likelihood to induce flakiness is impacted by complexity measures. Other features with a relevant IG are *Mystery Guest*, *Conditional Test Logic*, *Fire and Forget* and *Functional Decomposition* (for FlakeFlagger dataset), and *Eager Test*, the assert-related features (for both datasets), meaning that the presence of design flaws, either in production or test code, might provide indications of test flakiness.

Based on these results, we then verified the performance of Random Forest in terms of prediction capabilities. Table 8 presents data on the true positives, true negatives, false positives, false negatives, precision, recall, and F-Measure achieved on each project of the two datasets. The last rows (“Total”) report the results when considering all projects as a unique dataset.

**Table 8:** Results of the best classifiers for both datasets

Project	Tests	Flaky tests	TP	TN	FP	FN	Pr	R	F
iDFlakies			Random forest						
activiti	221	20	18	195	6	2	83%	90%	82%
admiral	2,082	5	3	2,066	11	3	21%	60%	31%
aletheia	46	3	3	40	3	0	50%	100%	66%
elastic-job-lite	564	3	2	554	7	1	22%	66%	33%
fastjson	544	12	8	530	2	4	75%	70%	70%
hadoop	12,838	58	36	12,766	14	22	77%	62%	66%
http-request	309	28	25	280	1	3	96%	90%	91%
incubator-dubbo	1,768	20	8	1,736	12	12	41%	40%	37%
java-websocket	135	27	26	92	16	1	63%	96%	75%
pippo	240	5	5	230	5	0	90%	100%	93%
querydsl	1,926	3	0	1,920	3	3	0%	0%	0%
struts	2,577	4	4	2,571	2	0	87%	100%	91%
wildfly	982	38	30	937	7	8	86%	79%	80%
Total	24,233	226	156	23,937	69	70	69%	69%	68%
FlakeFlagger			Random forest						
achilles	1,053	4	2	1,049	0	2	100%	50%	66%
activiti	169	16	5	141	12	11	25%	25%	23%
alluxio	186	122	117	60	4	5	97%	96%	97%
ambari	294	52	47	241	1	5	98%	90%	93%
elastic-job-lite	521	3	0	518	3	1	0%	0%	0%
hbase	368	121	105	233	14	16	89%	87%	87%
hector	121	33	26	75	13	7	76%	81%	74%
httpcore	524	15	8	503	6	7	50%	60%	53%
http-request	161	18	13	132	11	5	55%	75%	61%
incubator-dubbo	1,681	18	11	1,658	5	7	76%	65%	68%
java-websocket	107	21	20	86	0	1	100%	96%	98%
logback	655	15	3	637	3	12	50%	20%	28%
ninja	352	16	16	330	6	0	81%	100%	88%
okhttp	782	108	70	565	109	38	39%	65%	48%
orbit	26	4	2	20	2	2	50%	50%	50%
spring-boot	1,634	82	61	1,542	10	21	87%	74%	79%
undertow	48	6	2	39	3	4	40%	33%	26%
wro4j	1,103	16	3	1,084	3	13	14%	15%	12%
Total	9,785	670	446	8,957	158	224	74%	66%	70%

The first thing to discuss is concerned with the fact that, for both the datasets, we could not produce results for all individual projects. By diagnosing the reasons behind the failures of the model, we identified a main factor. On 37 projects of iDFlakies dataset and one project of FlakeFlagger dataset, the number of flaky tests was equal to one. This caused a training error, as the balancing algorithm failed because of the lack of instances to use when generating artificial elements.

The observations above already let us to point out a limitation in the use of machine learning for flaky test prediction. According to our data, there are cases where the unbalance problem is such that it is not even possible to train a machine learning model. On the one hand, this is a common limitation of machine learning applied to software engineering tasks (Azeem et al. 2019; Hall et al. 2011). On the other hand, our results point out the need for more specialized software engineering mechanisms to deal with peculiar properties of test flakiness: as an example, the use of cross-project models might be taken into consideration.

The inability to execute all models had an impact on the amount of our analysis. We could consider 13 projects of the iDFlakies dataset (for a total of 226 flaky tests on 24,232 test cases) and 18 projects of the FlakeFlagger dataset (for a total of 670 flaky tests on 9,785 test cases).

Looking at the performance obtained on the individual projects of the iDFlakies dataset, another interesting observation could be made. In one case, i.e., on the querydsl project, the machine learner behaved as a pessimistic classifier, predicting the non-flakiness of all test cases. This was clearly due to the few flaky test instances available in the dataset. Once again, this result seems to suggest that the balancing operations that might be reasonably performed might still be not enough. For this reason, alternative solutions to the prediction might be worth to explore.

In cases where the model could be built, the performance was reasonable and ranged between 31% and 93% of F-Measure. Diving into these projects, it is worth observing the presence of five projects, i.e., admiral, aletheia, elastic-job-lite, pippo and struts, that had a low amount of flaky tests but for which the model could still be built. To further understand the differences between these cases and the previously discussed one, we manually looked at the test code of the projects and the values for each feature. In particular, the first author examined the code and attempted to identify patterns that might explain why the model could be actually built. While the replication of such a qualitative analysis on a larger sample would be desirable, we could conjecture that in two projects the *diversity* of flaky cases was lower than the one of the project where the model could not be built. More specifically, the flaky tests of these projects belong to single test suites. The metric values computed on the test suites and the corresponding production classes are similar, in terms of lines of code and other design metrics. On the one hand, this is reasonable since these tests have been likely developed by the same developer, following the same design approach. On the other hand, some of our metrics aim at capturing aspects connected to the entire class, e.g., the TLOC metric: this implies that the value of some metrics is exactly the same, since test cases belong to the same class. As such, the balancing operation produced instances that, despite being artificial, could still be representative because derived from similar metric profiles. Such a rudimentary analysis seems to suggest that more comprehensive conceptual frameworks able to suggest when to use machine learning for flaky test prediction might be worth to devise.

Turning our attention to the FlakeFlagger dataset, we can observe that there is only one project where the number of true positives was zero, i.e., elastic-job-lite Besides this case, we could observe that the performance is almost always good, except for four projects in which the F-Measure does not even reach 50%. When putting all projects together, the number of true positives was high (446) and the number of false positives was low (158), with the performance metrics ranging from 66% to 74%.

In conclusion, our results provide two main insights. First, a fully static approach could reach high levels of accuracy in situations where the number of flaky tests is large enough or their diversity is low enough to ensure the learning of their characteristics. Second, there exist projects for which the use of machine learning does not look reasonable: further research effort should be spent to investigate when to use machine learning or to complement it with heuristic approaches that could assist when learning is not a suitable option. 
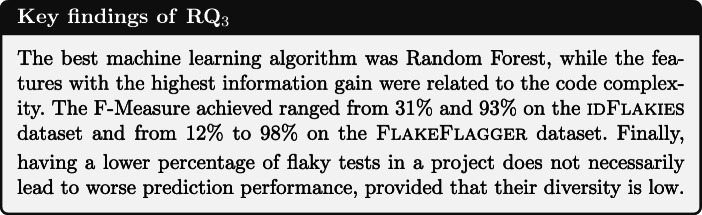


## RQ_4_ - Comparing the performance of the static approach with existing baselines

Our last research question aimed at comparing the performance of the static flaky test predictor with the currently existing baselines. This section reports on the methodological choices done and the results achieved.

### Research methodology

To address **RQ**_***4***_, we had to compare our fully static approach with existing baselines. To avoid threats to construct validity due to the re-implementation of the baselines, we decided to only focus on the FlakeFlagger dataset, which also provided data concerned with three baseline approaches such as (1) FlakeFlagger (Alshammari et al. 2021); (2) the textual-based approach proposed by Pinto et al. (Pinto et al. 2020), which we refer to as Vocabulary in the remainder of this section; and (3) the combination of the two (Alshammari et al. 2021), which we refer to as Combined in this section. Based on this methodological decision, we therefore decided not to consider the iDFlakies dataset in the context of **RQ**_***4***_.

More specifically, the data available pertain to the metrics used by the baseline approaches, namely the predictors employed to feed FlakeFlagger, Vocabulary, and Combined. On this basis, we could then proceed with the empirical comparison. To enable a fair comparison, we re-executed the same pipeline applied in **RQ**_***3***_ on the original features that have been released by Alshammari et al. (Alshammari et al. 2021). As such, we applied the vif function and computed the information gain (Quinlan 1986) to discard metrics not providing any gain. Afterwards, we trained a Random Forest algorithm—the choice was the result of a benchmark study where we experimented with multiple learning algorithms and under-/over-sampling strategies against the baseline data, finding that Random Forest combined with SMOTE was the best option to use to train the baselines. We then executed the models, collecting their performance and comparing them with our approach in terms of the same evaluation metrics employed in **RQ**_***3***_, i.e., precision, recall, and F-Measure. Finally, the Nemenyi test was applied to assess the statistical significance of the results achieved.

### Analysis of the results

Table 9 reports the information gain of each baseline feature in the FlakeFlagger dataset (Alshammari et al. 2021). To ease the comparison, we also reported the information gain data of our approach.

**Table 9:** List of features not excluded by the VIF analysis and with an information gain (IG) higher 0.001 for FlakeFlagger, Vocabulary approach, combined approach, and our model

Static approach		FlakeFlagger		
Features	IG	Features	Type	IG
Halstead Vocabulary	0.1727	Execution Time	FlakeFlagger	0.1414
Assertion Density	0.0539	Project Source Lines Covered	FlakeFlagger	0.0869
CBO	0.0359	Project Source Classes Covered	FlakeFlagger	0.0790
TLOC	0.0284	Covered Lines	FlakeFlagger	0.0400
Mystery Guest	0.0157	Covered Changes (past 500 commits)	FlakeFlagger	0.0328
McCabe	0.0133	Test Length	FlakeFlagger	0.0299
LCOM5	0.0128	Covered Changes (past 10000 commits)	FlakeFlagger	0.0258
Assertion Roulette	0.0107	Covered Changes (past 75 commits)	FlakeFlagger	0.0253
Conditional Test Logic	0.0076	Covered Changes (past 100 commits)	FlakeFlagger	0.0249
Eager Test	0.0066	Covered Changes (past 50 commits)	FlakeFlagger	0.0231
Fire and Forget	0.0013	mtfs	Token	0.0227
Functional Decomposition	0.0011	tfs	Token	0.0217
		External Library	FlakeFlagger	0.0188
		tachyon	Token	0.1716
		for	Token	0.0162
		Covered Changes (past 10 commits)	FlakeFlagger	0.0148
		fileid	Token	0.0132
		create	Token	0.0128
		int	Token	0.0128
		ioexception	Token	0.0126
		master	Token	0.0124
		writetype	Token	0.0120
		testutils	Token	0.0117
		assertthat	Token	0.0112
		tachyonfile	Token	0.0110
		throws	Token	0.016
		createbytefile	Token	0.0101
		Fire and Forget	FlakeFlagger	0.0101
		client	Token	0.0099
		Number of Assertions	FlakeFlagger	0.0097
		invalidpathexception	token	0.0095
		testfile	Token	0.0094
		that	Token	0.0088
		Covered Changes (past 5 commits)	FlakeFlagger	0.0087
		filealreadyexistexception	Token	0.0085
		file	Token	0.0083
		should	Token	0.0081
		cluster	Token	0.0081
		createfile	Token	0.0079
		Mystery Guest	FlakeFlagger	0.0078
		Resource Optimism	Token	0.0077
		new	Token	0.0071
		return	Token	0.0071
		asserttrue	Token	0.0069
		increasing	Token	0.0068
		null	Token	0.0067
		then	Token	0.0065
		throws	Token	0.0064
		thenreturn	Token	0.0064
		already	Token	0.0063
		true	Token	0.0063
		mkdir	Token	0.0061
		cli	Token	0.0060
		conf	Token	0.0060
		if	Token	0.0060
		Covered Changes (past 25 commits)	FlakeFlagger	0.0058

According to the data shown in the table, we could provide two main observations. First, we could confirm once again the role of code complexity. Indeed, among the most informative features considered by us and the baselines, we found both static and dynamic metrics related to complexity. For instance, features like execution time, test length, or number of external libraries are among the most relevant metrics. The role of complexity is also partially visible when looking at the tokens considered within the approach by Pinto et al. (Pinto et al. 2020). Indeed, terms like for or cli (the command line interface) suggest that the fact that a test performs complex tasks is an indication of flakiness. In addition, the most informative terms are connected to the management of files. As the reader might notice, the vast majority of the textual features in Table 9 pertain to exceptions (e.g., throws, ioexception, invalidpathexception, etc.) or to the creation of files (e.g., mkdir, createfile, createbytefile, etc.). Elaborating on the relevance of file-related terms, it may be reasonable to believe that an approach based on vocabulary is particularly suitable to identify flaky tests whose root cause depends on the sub-optimal management of files—this aspect might be interesting to consider in further experimentations on root cause classification.

In the second place, it is worth commenting on the fact that some features have different information gain when considered in our approach and in the baseline ones. Test smells are the main example. According to Alshammari et al. (Alshammari et al. 2021), *“none of the test smells [...] collected had a strong information gain, which may indicate that test smells are not well-correlated with test flakiness”*. Indeed, all the test smells appeared in the bottom of the ranked list of the baselines. In our case, the situation is slightly different: while the test smells scored lower than other features, their contribution seems to be comparable, hence possibly influencing test flakiness.

Such a difference could be explained by two factors. On the one hand, the static metrics could have less relevance than the dynamic ones when considered together. In other terms, the weight of the static features might be lower when dynamic information are available, hence leading these metrics to lose significance. On the other hand, Alshammari et al. (Alshammari et al. 2021) computed test smells in a different manner. As explained by the original authors, their goal was to *“not precisely detect test smells [...] but rather, to find features that may be representative of flaky tests”*. For this reason, they *“decided to expand [the] definition of many of these smells to be inclusive of all code executed by a test, rather than just the code contained in the test method body itself”*. In other terms, this detection mechanism aims at maximizing the recall, compromising the precision. As a consequence, the study by Alshammari et al. (Alshammari et al. 2021) might include a number of false positive test smell instances that could have biased the information gain computation. Our mechanism, instead, is based on a test smell detector that aims at optimizing the compromise between precision and recall (Pecorelli et al. 2020), hence providing a lower amount of false positives. Based on these observations, we argue the existence of a relation between test smells and flaky tests that might be worth to further explore—this relation was indeed partially confirmed by Camara et al. (Camara et al. 2021a), other than theorized in previous work (Palomba 2019).

**Fig. 4:**
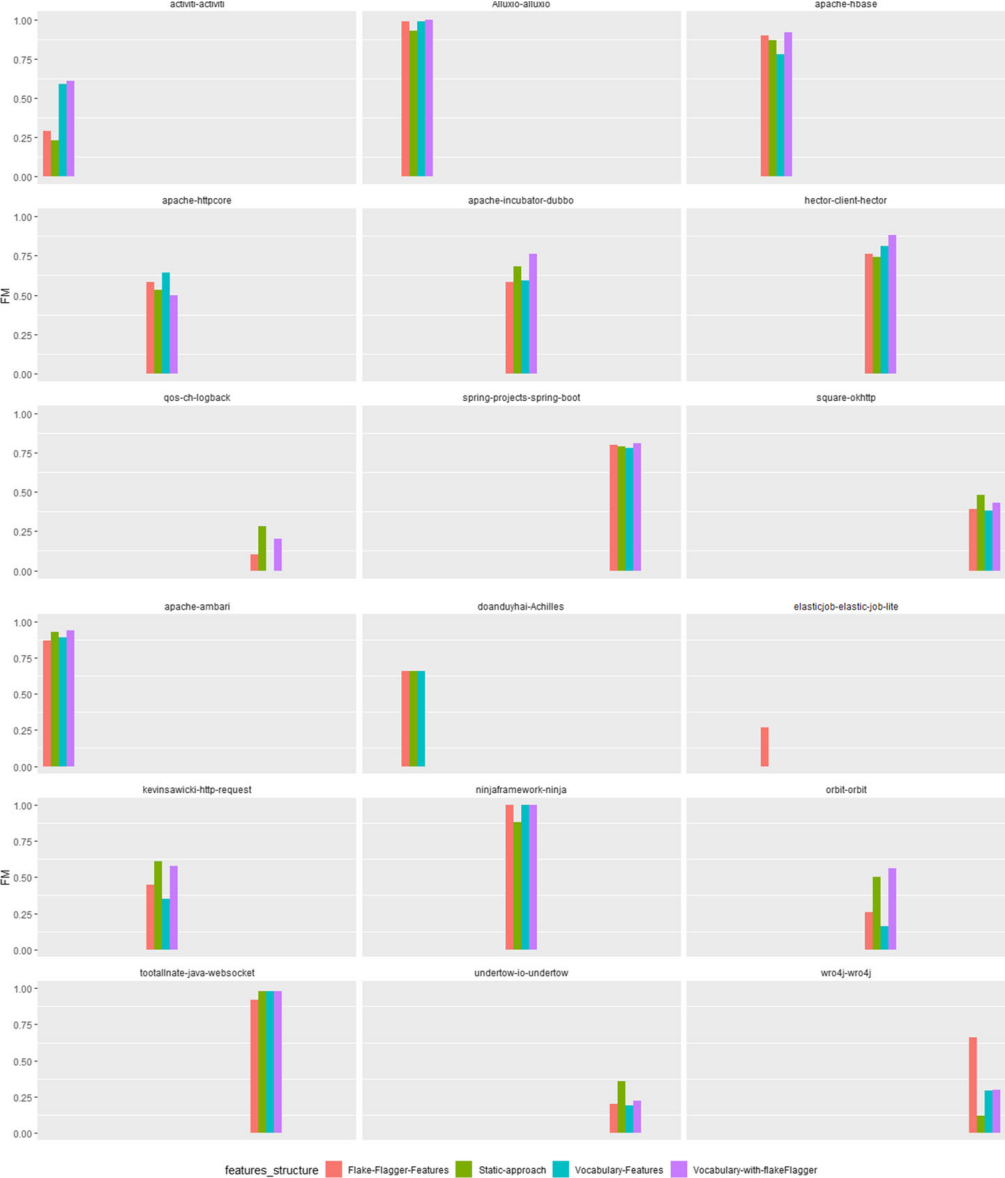
Barplot of the F-Measure achieved for each project when comparing the baselines to our static approach. The orange color represents FlakeFlagger, the green color represents our static approach, the blue color represents the Vocabulary, and the purple color represents the Combined

Table 10 reports the results obtained by the three baselines, showing the true positives, true negatives, false positives, false negatives, precision, recall, and F-Measure for each project and for the entire dataset. To ease the comparison, we also reported the results of our static approach. In addition, for a visual understanding of the results, Figure 4 depicts barplots of the F-Measure values obtained for each project by the experimented models.

**Table 10:** **RQ**_***4***_. Comparison between our model and the existing flaky test prediction models against the FlakeFlagger dataset

Project	TP	TN	FP	FN	Pr	R	F	TP	TN	FP	FN	Pr	R	F
	FlakeFlagger							Vocabulary approach						
achilles	2	1,049	0	2	100%	50%	66%	2	1,049	0	2	100%	50%	66%
activiti	5	143	10	10	31%	30%	29%	11	146	7	5	54%	70%	59%
alluxio	122	63	1	0	99%	100%	99%	121	64	0	1	100%	99%	99%
ambari	44	237	5	8	92%	84%	87%	43	241	1	9	97%	83%	89%
elastic-job-lite	1	515	3	2	25%	33%	27%	0	518	0	3	0%	0%	0%
hbase	110	236	11	11	91%	90%	90%	95	223	24	26	79%	78%	78%
hector	27	79	9	36	73%	81%	76%	26	83	5	7	87%	80%	81%
httpcore	12	496	13	3	48%	80%	58%	10	502	7	5	59%	75%	64%
http-request	11	127	16	7	39%	65%	45%	6	140	3	12	45%	30%	35%
incubator-dubbo	9	1,662	1	9	76%	50%	58%	10	1,661	2	8	71%	55%	59%
java-websocket	19	85	1	2	96%	91%	92%	20	86	0	1	100%	96%	98%
logback	1	636	4	14	10%	10%	10%	0	636	4	15	0%	0%	0%
ninja	16	336	0	0	100%	100%	100%	16	336	0	0	100%	100%	100%
okhttp	45	603	70	64	41%	41%	39%	33	650	23	76	58%	30%	38%
orbit	3	19	3	1	25%	30%	26%	2	21	1	2	15%	20%	16%
spring-boot	61	1,544	8	21	90%	74%	80%	59	1,544	8	23	88%	72%	78%
undertow	2	40	2	4	20%	50%	20%	1	40	2	5	33%	14%	19%
wro4j	1	1,086	1	15	50%	50%	66%	4	1,087	0	72	40%	25%	29%
Total	448	9,002	112	222	80%	66%	72%	428	9,006	108	242	80%	63%	70%
	Combined approach							Static approach						
achilles	0	1,049	0	0	0%	0%	0%	2	1,049	0	2	100%	50%	66%
activiti	11	147	6	5	56%	70%	61%	5	141	12	11	25%	25%	23%
alluxio	122	64	0	0	100%	100%	100%	117	60	4	5	98%	90%	93%
ambari	47	242	0	5	100%	90%	94%	47	241	1	5	98%	90%	93%
elastic-job-lite	0	518	0	3	0%	0%	0%	0	518	3	1	0%	0%	0%
hbase	112	238	9	9	92%	92%	92%	105	233	14	16	89%	87%	87%
hector	28	85	3	5	92%	86%	88%	26	75	13	7	76%	81%	74%
httpcore	9	501	8	6	44%	65%	50%	8	503	6	7	50%	60%	53%
http-request	10	140	3	8	70%	55%	58%	13	132	11	5	55%	75%	61%
incubator-dubbo	12	1,661	2	6	91%	70%	76%	11	1,658	5	7	76%	65%	68%
java-websocket	20	86	0	1	100%	96%	98%	20	86	0	1	100%	96%	98%
logback	2	638	2	13	50%	13%	20%	3	637	3	12	50%	20%	28%
ninja	16	336	0	0	100%	100%	100%	16	330	6	0	81%	100%	88%
okhttp	35	660	13	74	74%	31%	43%	70	565	109	38	39%	65%	48%
orbit	2	21	1	2	66%	50%	56%	2	20	2	2	50%	50%	50%
spring-boot	62	1,544	8	20	89%	75%	81%	61	1,542	10	21	87%	74%	79%
undertow	1	40	2	5	33%	16%	22%	2	39	3	4	40%	33%	26%
wro4j	3	1,087	0	13	100%	18%	30%	3	1,084	3	13	14%	15%	12%
Total	463	9,057	57	207	89%	68%	77%	446	8,957	158	224	74%	66%	70%

The table shows true positives, true negatives, false positives, false negatives, precision, recall, and F-Measure for each project and for the entire dataset. We report the results of both our static approach and the techniques already presented in the literature to facilitate comparison

Analyzing the results obtained for the entire dataset (row “Total”), we could first observe that the number of true positives of our approach is slightly lower with respect to the one of FlakeFlagger (446 vs 448) and Combined (446 vs 463), but higher to the one of Vocabulary (446 vs 423). Elaborating on these results, we could argue that it is reasonable to expect to identify less true positives, overall, since our approach is not boosted with dynamic features that would provide orthogonal pieces of information. Nonetheless, we could still observe similar levels of accuracy, especially when considering recall: this is indeed higher when compared to Vocabulary (66% vs 63%), equal to FlakeFlagger (66% vs 66%) and only slightly lower than Combined (66% vs 68%). From a practical perspective, these results imply that a similar amount of *actual* flaky tests can be identified in a more efficient manner by just looking at the design of test cases. The similar recall is payed in terms of precision: our approach outputs more false positives, overall. Nonetheless, the lower precision is not visible on all individual projects.

When looking at the results achieved on the individual projects, some considerations can be made. First, we could notice some complementarity between the experimented approaches. There are indeed cases where our approach cannot identify any flaky test, while the baselines can, and viceversa. This is, for instance, the case of the activiti, where the static approach performed worst than all other baselines. This project makes available a lightweight open-source business process management platform. In doing so, the source code implements a data-driven client-server architecture where data are sent back and forth to be verified. The corresponding tests are therefore called to verify that the data exchange processes work fine. By nature, the flakiness of these test cases might be more easily identified using dynamic or textual features: the former could help pinpointing edge cases through data-flows analysis, while the latter might exploit peculiar terms connected to the sub-optimal use of network protocols. On the contrary, the static metrics considered by our approach might not be effective in this case because none of them explicitly target the properties of source code. As a consequence, the baseline approaches tend to work better than ours.

On the other hand, let consider the logback project, which implements a framework to log Java code. In this case, the operations performed in the source code are mostly related to the management of files, e.g., by adding log statements to existing Java files. The corresponding test cases are therefore responsible to verify the correctness of such a file management. The detection of test flakiness, in this case, seems to be more connected to the static profile of a test, for instance to the way it handles the communication with files. This is a likely reason that makes our approach better than FlakeFlagger, other than the possible imprecision that the baseline has when computing certain static properties of source code, like test smells. Perhaps more interestingly, Vocabulary reached 0% precision and recall on this project, acting as a pessimistic classifier. We looked deeply into this case to understand the reason why an approach that mostly relies on file-related features failed so evidently. While we could not determine the exact reasons behind this failure, we noticed that the lack of natural language normalization might have impacted the performance of Vocabulary. Indeed, most file-related terms are taken as they are, even when different terms have the same (or similar) meaning. For instance, the source code of the logback project makes use of terms such as file and resilientfileoutputstream, file_header, file_footer, and others. While the terms actually refer to various specific properties or actions performed on files, a fully textual approach might not properly assess the likelihood of test flakiness because of the many different terms associated to the same potential issue arising with the management of files. In this sense, further improvements of the Vocabulary approach that take text normalization into account might be worth to explore.

There are, however, some exceptions to this discussion. In some cases the flaky tests can be predicted with a similar accuracy independently from the source of information exploited - for instance, in the cases of alluxio or ninja. Likely, this is due to the fact that either the static or dynamic metrics can capture the relevant aspects that may lead to the flakiness prediction.

To further elaborate on the complementarity among the experimented techniques, we conducted an additional analysis focused on understanding the overlap among them. Given two prediction models m_*i*_ and m_*j*_, we computed (1) the amount of flaky tests correctly predicted by both m_*i*_ and m_*j*_ and (2) the amount of flaky tests correctly predicted by m_*i*_ only and missed by m_*j*_. In addition, given the four experimented prediction models m_*i*_, m_*j*_, m_*k*_, and m_*p*_ we computed (1) the amount of flaky tests correctly predicted by all models and (2) the amount of flaky tests correctly predicted by m_*i*_ only and missed by m_*j*_, m_*k*_, and m_*p*_. Such an analysis could provide insights into the complementarity of the experimented techniques, other than assessing the actual value of our model with respect to the baselines.

The overlap results are reported in Table 11. The findings indicate a clear trend. When comparing our model with the baselines, we could observe that 72% of the correct predictions are in common. This means that the vast majority of the flaky tests can be detected independently from the model exploited. The complementarity is limited to the remaining portion of flaky tests. Our model can, for instance, identify 14% of flaky tests that FlakeFlagger cannot detect, and viceversa. This suggests that the cases of activiti and logback previously discussed represent exceptions to the general trend, while in most cases our model provides the same predictions as baselines that exploit additional dynamic or textual information.

**Table 11:** The overlap results. First, we reported the results obtained by comparing our model with the baselines, then we reported the results obtained by comparing the baselines with each other. Finally, the results obtained by comparing the values predicted correctly by a single model that were not predicted by the other three are reported

Static vs. FlakeFlagger		
Static _*corr*_ ∩ FlakeFlagger _*corr*_	Static _*corr*_ ∖ FlakeFlagger _*corr*_	FlakeFlagger _*corr*_ ∖ Static _*corr*_
72%	14%	14%
Static vs. Vocabulary		
Static _*corr*_ ∩ Vocabulary _*corr*_	Static _*corr*_ ∖ Vocabulary _*corr*_	Vocabulary _*corr*_ ∖ Static _*corr*_
72%	16%	12%
Static vs. Combined		
Static _*corr*_ ∩ Combined _*corr*_	Static _*corr*_ ∖ Combined _*corr*_	Combined _*corr*_ ∖ Static _*corr*_
72%	14%	14%
FlakeFlagger vs. Vocabulary		
FlakeFlagger _*corr*_ ∩ Vocabulary _*corr*_	FlakeFlagger _*corr*_ ∖ Vocabulary _*corr*_	Vocabulary _*corr*_ ∖ FlakeFlagger _*corr*_
70.7%	16.4%	12.9%
FlakeFlagger vs. Combined		
FlakeFlagger _*corr*_ ∩ Combined _*corr*_	FlakeFlagger _*corr*_ ∖ Combined _*corr*_	Combined _*corr*_ ∖ FlakeFlagger _*corr*_
78.8%	8.6%	12.7%
Vocabulary vs. Combined		
Vocabulary _*corr*_ ∩ Combined _*corr*_	Vocabulary _*corr*_ ∖ Combined _*corr*_	Combined _*corr*_ ∖ Vocabulary _*corr*_
82.6%	5.1%	12.3%
Static _*corr*_ ∖ (FlakeFlagger _*corr*_ ∪ Vocabulary _*corr*_ ∪ Combined _*corr*_)	FlakeFlagger _*corr*_ ∖ (Static _*corr*_ ∪ Vocabulary _*corr*_ ∪ Combined _*corr*_)	
15.5%	15.7%	
Vocabulary _*corr*_ ∖ (Static _*corr*_ ∪ FlakeFlagger _*corr*_ ∪ Combined _*corr*_)	Combined _*corr*_∖ (Static _*corr*_ ∪ FlakeFlagger _*corr*_ ∪ Vocabulary _*corr*_)	
13.2%	17.4%	
(Static _*corr*_ ∩ FlakeFlagger _*corr*_ ∩ Vocabulary _*corr*_ ∩ Combined) ∖ (Static _*corr*_ ∪ FlakeFlagger _*corr*_ ∪		
Vocabulary _*corr*_ ∪ Combined _*corr*_)		
38.2%		

Besides the relation between our model and the baselines, our analysis also indicates that the discussion is similar when comparing the other models against each other. Table 11 indeed reports that most of the flaky tests can be correctly identified by two baselines, with a limited amount of flaky tests detected by only one of them.

The results are further confirmed when looking at the bottom of Table 11. When studying the amount of flaky tests correctly identified by all approaches, we could see that this happened in 38% of the cases. The contributions of the individual models reach up to 17% in the case of Combined.

To conclude, the observations above—especially those related to the overlap analysis—seem to reinforce and extend what discovered in **RQ**_***3***_: a fully static approach that does not require expensive dynamic or textual computation can provide insights into the flakiness of test cases with an accuracy close (or higher, in some cases) of more sophisticated baselines. 
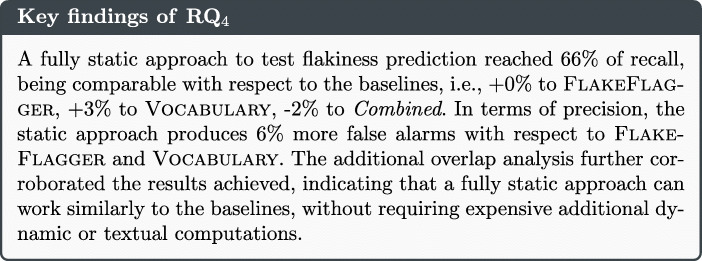


## Threats to validity

When it comes to the limitations of the study, there are some factors that might have biased our conclusions. This section discusses these factors and the mitigation strategies applied to limit their influence on our results.

### Construct validity

The main threat related to the relationship between theory and observation is concerned with possible imprecision in the data used in the study. We relied on publicly available sources built in the context of previous researches (Alshammari et al. 2021; Lam et al. 2019) and that have been already used and validated. This makes us confident of the reliability of the datasets; yet, we cannot exclude imprecision, especially in terms of the flaky tests identified, e.g., some tests might have not exposed their unreliability over the multiple executions performed by the authors of the datasets. In this sense, further replications conducted on different datasets might be worth to increase the confidence on the validity of our results.

Another discussion point concerns with the computation of the independent variables through automated tools. We are aware of the possible noise that might be introduced, for instance in terms of false positive code and test smells. Yet, we had to necessarily accept this limitation, as our study targeted large datasets for which a manual detection process was infeasible. To partially mitigate this threat, we selected well-established tools that have been previously evaluated, showing good accuracy. In addition, we defined independent variables by computing metrics on either production or test code, while additional analyses might consider the effects of computing metrics on both of them. For instance, some code smells (e.g., *Complex Code*) might be a potentially relevant indicator of test flakiness. Further investigations on this matter are part of our future research agenda.

When computing independent variables, we had to link test classes to the corresponding production classes. To this aim, we relied on a pattern matching approach relying on naming conventions. Multiple observations should be made on this choice. In the first place, the choice of using it comes from the good compromise between accuracy and scalability it guarantees. As already mentioned in Section 4, alternative approaches based on more complex algorithms, e.g., static and dynamic slicing (Qusef et al. 2013), are typically more effective but poorly scalable on a large scale. In our study, we accepted the limitations of the pattern matching approach with the aim of conducting a larger scale evaluation. However, we took some precautions. In particular, the approach may output false positive links in cases where two or more production classes have identical names, but different paths. Dealing with these cases was not necessary in our case, as there were no cases of production classes with identical names but different paths. Nonetheless, replications of our study on different systems may need to consider this potential concern to improve the linking capabilities of the pattern matching approach.

Finally, in the context of **RQ**_***4***_, we decided to only focus on the FlakeFlagger dataset. While this decision let us reduce the amount of data, it allowed us to avoid the re-implementation of the baselines. Being not the original authors of those approaches, our re-implementation could have introduced bias, affecting the validity and fairness of the comparison.

### Conclusion validity

Threats to conclusion validity are related to the relationship between treatment and outcome. As for the statistical methods employed in **RQ**_***2***_, we selected the Generalized Linear Model after verifying its suitability for our purpose, e.g., its ability to deal with dichotomous variables. In addition, to ensure that the model did not suffer from multi-collinearity, we applied a stepwise procedure, using the vif function, aimed at discarding non-relevant independent variables. These procedures followed established guidelines (O’brien 2007), making us confident of the validity of the conclusions drawn.

With respect to the machine learning exercises conducted in **RQ**_***3***_ and **RQ**_***4***_, we benchmarked multiple learning algorithms, trained using different under- and over-sampling strategies, in order to identify the best performing one. The performance of Random Forest in terms of F-Measure were better than the other models, overall, as shown by the Nemenyi test. Our online appendix (Pontillo et al. 2022) includes the data and analysis scripts used to reach this conclusion. Moreover, the quantitative results have been backed-up with the use of appropriate statistical tests and more qualitative, manual analyses conducted to verify the rationale behind some of the observed findings.

Another relevant discussion point concerns with the validation strategy used to reach conclusions. In our study, we work in the context of a cross-validation scenario. Nonetheless, we are aware of the possible limitations coming from this design choice: flakiness data are indeed likely to be time-sensitive and a validation strategy accounting for this aspect might substantially vary the interpretation of the performance metrics. There are, however, two main observations to make in this respect.

First and foremost, previous work on flaky test prediction, i.e., all the experimented baselines (Alshammari et al. 2021; Lam et al. 2019; Pinto et al. 2020), employed a cross-validation procedure. As such, a variation of the validation strategy would not have allowed us to perform a fair, precise comparison to quantify the value of statically-computable metrics with respect to the others previously proposed in literature.

Perhaps more importantly, a time-sensitive validation would have required a dedicated research design, other than expensive computations due to the mining of flaky tests over the history of the considered software systems. More particularly, while the datasets employed in the study provide information on the commits where a flaky test was detected, the mining procedure followed to identify those flaky tests was not meant to conduct a time-sensitive validation and might therefore require some tuning/adjustments. For the sake of concreteness, let us consider the case of the commit 7e3801e19fb43183c59607663ebd53c27a95cf77 of the WRO4J project, where the test case named testbourboncssprocessor.shouldbethreadsafe was detected as flaky. By analyzing this case further, we found out that the commit did not modify the test nor the associated production class (i.e., the class named bourboncssprocessor). In addition, the modified classes did not have any structural relation with neither the production nor test class. Yet, the flakiness of the test emerged. In other terms, the flakiness affecting the test manifested itself independently from the actions performed by developers within the commit. This implies that the test might have possibly been flaky even in previous commits of the project, despite not being detected. The example has two main implications. First, novel strategies to identify flakiness-inducing commits should be devised, as they should not only rely on the information coming from an individual commit of the change history (as the flakiness might have been previously emerged), but rather should mark flakiness by also looking at the specific change history of tests (e.g., starting from the emergence of a flaky test, they may traverse in reverse order the commits until the last modification of the test). Second, the information available in current datasets might potentially lead to biased observations when flaky test prediction models are experimented in a time-sensitive fashion, as they were not collected by explicitly considering the many perils of mining flaky test data. For these reasons, we believe that such an analysis would require a brand new set of research questions, methodology, and analyses, and is, therefore, out of the scope of our current submission.

Finally, it is worth discussing about the relation between the performance observed when executing our model and complexity. Throughout the analysis of the results we have highlighted the role of complexity metrics to discriminate the flakiness of a test case. This may potentially lead to a practical limitation of our approach: there is no guarantee that fixing a flaky test would reduce its complexity, which is apparently what is useful to identify them, whereas dynamic metrics would supposedly find differences (e.g., different coverage). In this case, the approach would potentially not be useful to developers that would get false positives from the model once their flaky tests have been fixed. There are two observations to make in this respect. First, it is reasonable to believe that the problem mostly pertains to code complexity metrics computed on production code. Indeed, while the complexity of the exercised code may provide hints to our prediction model, previous work (Lam et al. 2019; Luo et al. 2014) pointed out that the fixing of a flaky test often revolves around the modification of the test code only, hence increasing the risk of future misclassifications of our model. The same may not be immediately applicable to complexity metrics computed on test code: the likelihood of a fixing operation reducing test code complexity is higher, as any modification induces changes in terms of metrics. Our model relies on complexity metrics computed on both test and production code (see Table 1) and, according to the results achieved in **RQ**_***3***_, the Information Gain analysis revealed that the complexity of test code (as indicated by the McCabe metric) appeared to be important in both datasets. As such, the real-world capabilities of our model may be driven by multiple complexity metrics that capture aspects connected to both test and production code.

In any case, to further analyze the practical capabilities of our approach, we performed an additional analysis aiming at verifying the behavior of the model when applied before and after fixes to flaky tests. To this aim, we exploited the iFixFlakies dataset (Shi et al. 2019). In particular, in the context of their work, Shi et al. (Shi et al. 2019) opened 32 pull requests proposing to the contributors of the considered projects to integrate changes that would have fixed flaky tests of their applications. 23 of these pull requests were finally accepted and integrated. Shi et al. (Shi et al. 2019) also provided an online appendix reporting the results of the pull request analysis.^6^ We used this dataset to identify the flaky tests whose fixes were accepted by contributors and that are in common with our dataset - recall that we had to discard some tests or projects because of our requirement of detecting the production class associated with the test taken into account (see Section 4). Among the 23 cases of accepted pull requests, we could identify four cases suitable for the additional analysis. First and foremost, in all cases our model was able to correctly classify the flakiness of the tests before and after the fix. Analyzing the metric profile of the tests further, we could observe that in two cases the intensity of the *Eager Test* smell instances affecting the earlier version of the test was reduced during the fixing process. More importantly, the value of metrics such as WMC, RFC, MPC, and Halstead’s vocabulary was reduced, meaning that the fixes induced changes that had the effect of reducing the overall complexity of the code - hence, positively influencing the model’s capabilities. In the remaining two cases analyzed, we observed no variation in terms of test code metrics, yet the Halstead’s vocabulary metric value of the production code was reduced.

We are aware that the limited extent of the analysis does not allow us to generalize the results achieved. At the same time, the few cases analyzed seem to highlight some peculiarities of the flaky test fixing process: not only this leads to the removal of the flakiness, but also tends to induce variations in the metric profile of both test and production code, especially in terms of code complexity. This is the likely reason why our model could correctly discriminate the flakiness of test cases both before and after the fixes. Of course, further investigations should corroborate our initial findings - and further datasets should be developed so that these kind of analyses may be enabled.

### External validity

Threats to external validity regard the generalizability of the results. We conducted our study focusing on the iDFlakies and FlakeFlagger datasets (Alshammari et al. 2021; Lam et al. 2019), which are limited to open-source projects written in Java. In this respect, it is important to note that the projects have different scope and characteristics that allow us in part to mitigate this threat. While this is still a limitation of our study, there are two considerations to make. First, the vast majority of the datasets collecting information on flaky tests pertain to Java projects. This is the reason why we decided to focus on Java in the first place. This recalls the need for additional datasets targeting different programming languages: while some attempts have been made in the recent past (Gruber et al. 2021; Dutta et al. 2020), our work further remarks this need. In the second place, it is reasonable to believe that our approach might work when applied to other object-oriented applications, where the static metrics considered could be computed. Of course, an extension of this type would require additional investigations and instruments. For example, specialized code and test smell detectors have been proposed for Python (Vavrová and Zaytsev 2017; Wang et al. 2021), yet these target peculiar design issues arising in Python code. As such, replications of our work aiming at understanding the relation between Python-specific code/test smells and test flakiness should be devised before considering the effect of static indicators for flaky test prediction. In a similar vein, our approach could be experimented on other object-oriented programming languages. As for other types of programming languages (e.g., procedural ones), it is important to notice that the concepts used in our study can be adapted as well: code metrics and smells might be defined and detected in procedural languages as well (e.g., (de Almeida Filho et al. 2019)), hence making a wider application of our work potentially feasible. In any case, extensions like those mentioned above are part of our future research agenda.

An addition point concerns with the practical adoption of our approach. The methodology employed to link test to production classes naturally limits the applicability of the current version of the approach to the projects that actually employ naming conventions. Nonetheless, the choice of using the pattern matching approach does not necessarily influence the practical deployment of our approach. Developers interested in using our solution may indeed configure it so that the linking process is performed according to the standards/guidelines they normally apply to develop code, leading our approach to be fed with even more data. In other terms, the empirical choices applied in our study were taken to provide a larger-scale experimentation of the approach, but in a real-world case the availability of a stronger or *ad-hoc* linking solution might potentially lead to having larger datasets to train our model, which is supposed to further increase the performance reported in our paper.

## Conclusion, discussion, and future work

Test flakiness concerns with the non-determinism of test cases, which might lead developers to waste time in diagnosing source code, other than increasing the overall testing costs. While the most common approach to their detection is represented by the multiple re-execution of test cases, a number of recent studies proposed the adoption of machine learning approaches that could predict flaky tests in advance. Nonetheless, most of these artificial intelligence solutions require the computation of dynamic metrics, like code coverage, or the analysis of textual properties of test code. These still make the prediction exercise not scalable, possibly impacting their practicality.

In this paper, we conducted an empirical study to analyze whether and to what extent static metrics might be used to predict test flakiness. We selected features of different nature, including test and production code metrics and smells. First, we studied how these features correlate with test flakiness: this was done by analyzing both features individually and in combination. The promising results obtained from such an investigation allowed us to verify how the considered factors could be employed within machine learning solutions. Hence, we devised a fully static approach to test flakiness prediction. The empirical investigation aimed at (1) measuring the performance of the approach and (2) comparing them with those achieved by three baselines based on dynamic features, source code vocabulary, and their combination. This empirical study provided a number of notable findings:

- Code complexity metrics are the ones that differ the most between flaky and non-flaky tests. Not only this result was confirmed on both the considered datasets, but also when looking at the most relevant features employed by the fully static approach. This has two main implications. On the one hand, practitioners might use our findings to justify the adoption of instruments to take code complexity under control. On the other hand, more research on code complexity and how it affects test code quality might be worth to further elaborating instruments to support developers.
- When analyzing the value of the features used by our approach and by the baselines, we observed that some of them have a different weight. Particularly, while test smells were not deemed relevant for FlakeFlagger, they contributed to our approach in a comparable manner with respect to other features. This opens up new research opportunities into the relation between test smells and flakiness. Some research on the matter has been recently proposed (Camara et al. 2021a), yet we argue that more empirical investigations might be conducted to further understand how test code quality impacts the likelihood of test flakiness.
- A fully static approach to test flakiness prediction reaches comparable results with respect to the baselines—the F-Measures ranged from 17% to 99% on the two considered datasets. Perhaps more importantly, our approach has higher precision, hence representing a more practical solution for developers. While additional investigations into the matter are already part of our future research agenda, our results have already implications for researchers and practitioners. The former are called to devise and study novel, more powerful metrics that could contribute to the improvement of the flakiness prediction capabilities. The latter may rely on an approach that does not need dynamics computations to verify the quality and reliability of the test cases developed within their own organization. From a practical standpoint, the static nature of the experimented model would let it be run among the other continuous checks that developers normally do to verify the presence of regressions in newly committed code (Vassallo et al. 2020).
- Our study revealed some peculiarities of the flakiness data that might lead machine learning approaches to work differently. In particular, we identified the diversity of test cases as a relevant factor to even allow a machine learner to work. In addition, we also found some interesting complementarity between our approach and the baselines, which suggests that improvements are still possible. On the basis of these conclusions, we argue that the results of this paper might lead to further research on novel software engineering practices for flaky test prediction, namely instruments and methodologies that are aware of the flakiness data properties and may act accordingly, for instance by dynamically selecting the approach to use or the pre-processing steps to apply.

The output of this study represents the input of our future research agenda, which will be focused on further understanding the relation between static metrics (e.g., code complexity, code smells, or test smells) and test flakiness. In addition, we aim at conducting additional investigations on how to best configure and evaluate machine learning pipelines for the problem of flaky test prediction. Part of these investigations will also revolve around the problem of mining flakiness-inducing commit, which may enable further time-sensitive analysis of flaky test prediction models other than investigations into the flakiness detection and fixing process. Finally, we aim at devising novel artificial intelligence techniques that could combine existing instruments, other than recommending when to use a technique rather than another.

## Data Availability

The datasets generated and analysed during the current study are available as part of our online appendix on Figshare: 10.6084/m9.figshare.17080946.

## Footnotes

1 The iDFlakies dataset: https://sites.google.com/view/flakytestdataset/home.

2 The FlakeFlagger dataset: https://zenodo.org/record/4450723#.YXetWprP2Uk.

3 Available at: https://github.com/acmsigsoft/EmpiricalStandards.

4 https://www.r-project.org/

5 https://www.r-project.org/

6 Available at: https://sites.google.com/view/ifixflakies

## References

[CR1] Alshammari A, Morris C, Hilton M, Bell J (2021) Flakeflagger: Predicting flakiness without rerunning tests. In: ICSE 2021, IEEE, pp 1572–1584

[CR2] Association IS (1998) 829-1998 IEEE standard for software test documentation, Tech. rep., Technical report

[CR3] Azeem MI, Palomba F, Shi L, Wang Q (2019). Machine learning techniques for code smell detection: a systematic literature review and meta-analysis. Inf Softw Technol.

[CR4] Azhagusundari B, Thanamani AS (2013). Feature selection based on information gain. Int J Innov Technol Exploring Eng (IJITEE).

[CR5] Banko M, Brill E (2001) Scaling to very very large corpora for natural language disambiguation. In: Proceedings of the 39th annual meeting of the association for computational linguistics, pp 26–33

[CR6] Bell J, Kaiser G, Melski E, Dattatreya M (2015) Efficient dependency detection for safe java test acceleration. In: Proceedings of the 2015 10th joint meeting on foundations of software engineering, pp 770–781

[CR7] Bell J, Legunsen O, Hilton M, Eloussi L, Yung T, Marinov D (2018) Deflaker: automatically detecting flaky tests. In: ICSE 2018, IEEE, pp 433–444

[CR8] Bengio Y, Grandvalet Y (2004). No unbiased estimator of the variance of k-fold cross-validation. J Mach Learn Res.

[CR9] Bergstra J, Bengio Y (2012). Random search for hyper-parameter optimization. J Mach Learn Res.

[CR10] Bertolino A, Cruciani E, Miranda B, Verdecchia R (2021). Know your neighbor: fast static prediction of test flakiness. IEEE Access.

[CR11] Camara B, Silva M, Endo A, Vergilio S (2021) On the use of test smells for prediction of flaky tests. In: Brazilian symposium on systematic and automated software testing, pp 46–54

[CR12] Camara B, Silva M, Endo A, Vergilio S (2021) What is the vocabulary of flaky tests? an extended replication. pp 444–454

[CR13] Catolino G, Palomba F, Zaidman A, Ferrucci F (2019) How the experience of development teams relates to assertion density of test classes. In: ICSME 2019, IEEE, pp 223–234

[CR14] Chawla NV, Bowyer KW, Hall LO, Kegelmeyer WP (2002). Smote: synthetic minority over-sampling technique. J Artif Intell Res.

[CR15] Chidamber S, Kemerer C (1994). A metrics suite for object oriented design. IEEE TSE.

[CR16] Cordy M, Rwemalika R, Franci A, Papadakis M, Harman M (2022) Flakime: laboratory-controlled test flakiness impact assessment

[CR17] Daniel B, Jagannath V, Dig D, Marinov D (2009) Reassert: suggesting repairs for broken unit tests. In: ASE 2009, IEEE, pp 433–444

[CR18] de Almeida Filho FG, Martins ADF, da Silva Vinuto T, Monteiro JM, de Sousa ÍP, de Castro Machado J, Rocha LS (2019) Prevalence of bad smells in pl/sql projects. In: 2019 IEEE/ACM 27Th international conference on program comprehension (ICPC), IEEE, pp 116–121

[CR19] de Paulo Sobrinho EV, De Lucia A, de Almeida Maia M (2018). A systematic literature review on bad smells—5 w’s: which, when, what, who, where. IEEE Trans Softw Eng.

[CR20] dos Reis JP, e Abreu FB, de Figueiredo Carneiro G, Anslow C (2021). Code smells detection and visualization: a systematic literature review. Arch Comput Methods Eng.

[CR21] Dutta S, Shi A, Choudhary R, Zhang Z, Jain A, Misailovic S (2020) Detecting flaky tests in probabilistic and machine learning applications. In: Proceedings of the 29th ACM SIGSOFT international symposium on software testing and analysis, pp 211–224

[CR22] Eck M, Palomba F, Castelluccio M, Bacchelli A (2019) Understanding flaky tests: the developer’s perspective. In: ESEC/FSE 2019, pp 830–840

[CR23] Fowler M (2011) Eradicating non-determinism in tests. Martin Fowler Personal Blog. https://martinfowler.com/articles/nonDeterminism.html

[CR24] Fowler M (2018) Refactoring: improving the design of existing code. Addison-Wesley Professional

[CR25] Freund Y, Mason L (1999) The alternating decision tree learning algorithm. In: Icml, vol 99. Citeseer, pp 124–133

[CR26] Garson G (2012) Testing statistical assumptions. Asheboro NC: Statistical Associates Publishing

[CR27] Grano G, De Iaco C, Palomba F, Gall H (2020) Pizza versus pinsa: on the perception and measurability of unit test code quality. In: ICSME 2020, IEEE, pp 336–347

[CR28] Grano G, Palomba F, Gall H (2019) Lightweight assessment of test-case effectiveness using source-code-quality indicators IEEE TSE

[CR29] Greiler M, Van Deursen A, Storey MA (2013) Automated detection of test fixture strategies and smells. In: 2013 IEEE sixth international conference on software testing, verification and validation, IEEE, pp 322–331

[CR30] Gruber M, Fraser G (2022)

[CR31] Gruber M, Lukasczyk S, Kroiß F, Fraser G (2021) An empirical study of flaky tests in python. In: 2021 14Th IEEE conference on software testing, verification and validation (ICST), IEEE, pp 148–158

[CR32] Gyori A, Shi A, Hariri F, Marinov D (2015) Reliable testing: detecting state-polluting tests to prevent test dependency. In: Proceedings of the 2015 international symposium on software testing and analysis, pp 223–233

[CR33] Habchi S, Haben G, Papadakis M, Cordy M, Traon YL (2021)

[CR34] Haben G, Habchi S, Papadakis M, Cordy M, Le Traon Y (2021) A replication study on the usability of code vocabulary in predicting flaky tests. In: MSR 2021

[CR35] Hall T, Beecham S, Bowes D, Gray D, Counsell S (2011). A systematic literature review on fault prediction performance in software engineering. IEEE Trans Softw Eng.

[CR36] Han H, Wang WY, Mao BH (2005) Borderline-smote: a new over-sampling method in imbalanced data sets learning. In: International conference on intelligent computing, Springer, pp 878–887

[CR37] Han J, Kamber M, Pei J (2011). Data mining concepts and techniques third edition. Morgan Kaufmann Ser Data Manag Syst.

[CR38] Harman M, O’Hearn P (2018) From start-ups to scale-ups: Opportunities and open problems for static and dynamic program analysis. In: 2018 IEEE 18Th international working conference on source code analysis and manipulation (SCAM), pp 1–23. 10.1109/SCAM.2018.00009

[CR39] He H, Bai Y, Garcia EA, Li S (2008) Adasyn: adaptive synthetic sampling approach for imbalanced learning. In: 2008 IEEE international joint conference on neural networks (IEEE world congress on computational intelligence), IEEE, pp 1322–1328

[CR40] Ho TK (1995) Random decision forests. In: Proceedings of 3rd international conference on document analysis and recognition, vol 1. IEEE, pp 278–282

[CR41] Khomh F, Penta MD, Guéhéneuc YG, Antoniol G (2012). An exploratory study of the impact of antipatterns on class change-and fault-proneness. Empir Softw Eng.

[CR42] Kohavi R (1995) A study of cross-validation and bootstrap for accuracy estimation and model selection. In: Proceedings of the 14th international joint conference on artificial intelligence - volume 2, IJCAI’95. Morgan Kaufmann Publishers Inc., San Francisco, pp 1137–1143

[CR43] Koning AJ, Franses PH, Hibon M, Stekler HO (2005). The m3 competition: Statistical tests of the results. Int J Forecast.

[CR44] Kramer O (2016) Scikit-learn. In: Machine learning for evolution strategies, Springer, pp 45–53

[CR45] Lacoste F (2009) Killing the gatekeeper: introducing a continuous integration system. In: 2009 Agile conference, IEEE, pp 387–392

[CR46] Lam W, Oei R, Shi A, Marinov D, Xie T (2019) Idflakies: a framework for detecting and partially classifying flaky tests. In: ICST 2019, IEEE, pp 312–322

[CR47] Lam W, Winter S, Astorga A, Stodden V, Marinov D (2020) Understanding reproducibility and characteristics of flaky tests through test reruns in java projects. In: ISSRE 2020, IEEE, pp 403–413

[CR48] Lam W, Winter S, Wei A, Xie T, Marinov D, Bell J (2020). A large-scale longitudinal study of flaky tests. Proc ACM Prog Lang.

[CR49] Lambiase S, Cupito A, Pecorelli F, De Lucia A, Palomba F (2020) Just-in-time test smell detection and refactoring: the darts project. In: Proceedings of the 28th international conference on program comprehension, pp 441–445

[CR50] Lampel J, Just S, Apel S, Zeller A (2021) When life gives you oranges: detecting and diagnosing intermittent job failures at mozilla. In: 29Th ACM joint meeting on european software engineering conference and symposium on the foundations of software engineering, pp 1381–1392

[CR51] Luo Q, Hariri F, Eloussi L, Marinov D (2014) An empirical analysis of flaky tests. In: ESEC/FSE 2014, pp 643–653

[CR52] McCabe T (1976). A complexity measure. IEEE TSE.

[CR53] Memon A, Cohen M (2013) Automated testing of gui applications: models, tools, and controlling flakiness. In: ICSE 2013, IEEE, pp 1479–1480

[CR54] Micco J (2017) The state of continuous integration testing@ google. https://research.google/pubs/pub45880/

[CR55] Moha N, Guéhéneuc Y, Duchien L, Le Meur A (2009). Decor: a method for the specification and detection of code and design smells. IEEE TSE.

[CR56] Murillo-Morera J, Jenkins M (2015) A software defect-proneness prediction framework: a new approach using genetic algorithms to generate learning schemes. In: SEKE, pp 445–450

[CR57] Myers L, Sirois MJ (2004) Spearman correlation coefficients, differences between. Encycl Stat Sci :12

[CR58] Nelder J, Wedderburn R (1972). Generalized linear models. J R Stat Soc Ser A (Gen).

[CR59] Nemenyi PB (1963) Distribution-free multiple comparisons Princeton University

[CR60] Noble WS (2006). What is a support vector machine?. Nat Biotechnol.

[CR61] O’brien R (2007). A caution regarding rules of thumb for variance inflation factors. Qual Quant.

[CR62] Palomba F (2019) Flaky tests: problems, solutions, and challenges. In: BENEVOL

[CR63] Palomba F, Bavota G, Di Penta M, Fasano F, Oliveto R, De Lucia A (2018). On the diffuseness and the impact on maintainability of code smells: a large scale empirical investigation. Empir Softw Eng.

[CR64] Palomba F, Bavota G, Di Penta M, Oliveto R, Poshyvanyk D, De Lucia A (2014). Mining version histories for detecting code smells. IEEE Trans Softw Eng.

[CR65] Palomba F, Panichella A, Zaidman A, Oliveto R, De Lucia A (2017). The scent of a smell: an extensive comparison between textual and structural smells. IEEE Trans Softw Eng.

[CR66] Palomba F, Zaidman A, De Lucia A (2018) Automatic test smell detection using information retrieval techniques. In: 2018 IEEE international conference on software maintenance and evolution (ICSME), IEEE, pp 311–322

[CR67] Parry O, Kapfhammer GM, Hilton M, McMinn P (2021). A survey of flaky tests. ACM Trans Softw Eng Methodol (TOSEM).

[CR68] Pecorelli F, Catolino G, Ferrucci F, De Lucia A, Palomba F (2022). Software testing and android applications: a large-scale empirical study. Empir Softw Eng.

[CR69] Pecorelli F, Di Lillo G, Palomba F, De Lucia A (2020) Vitrum: a plug-in for the visualization of test-related metrics. In: AVI 2020, pp 1–3

[CR70] Pecorelli F, Palomba F, De Lucia A (2021) The relation of test-related factors to software quality: a case study on apache systems, vol 26

[CR71] Pecorelli F, Palomba F, Di Nucci D, De Lucia A (2019) Comparing heuristic and machine learning approaches for metric-based code smell detection. In: 2019 IEEE/ACM 27Th international conference on program comprehension (ICPC), IEEE, pp 93–104

[CR72] Perez A, Abreu R, van Deursen A (2017) A test-suite diagnosability metric for spectrum-based fault localization approaches. In: ICSE 2017, IEEE, pp 654–664

[CR73] Peruma A, Almalki K, Newman CD, Mkaouer MW, Ouni A, Palomba F (2020) Tsdetect: an open source test smells detection tool. In: Proceedings of the 28th ACM joint meeting on european software engineering conference and symposium on the foundations of software engineering, pp 1650–1654

[CR74] Pezze M, Young M (2008). Software testing and analysis: process, principles, and techniques.

[CR75] Pinto G, Miranda B, Dissanayake S, D’Amorim M, Treude C, Bertolino A (2020) What is the vocabulary of flaky tests?. In: MSR 2020, pp 492–502

[CR76] Pontillo V, Palomba F, Ferrucci F (2021) Toward static test flakiness prediction: a feasibility study. In: MaLTESQuE 2021, Association for Computing Machinery, New York, NY, USA, pp 19–24. 10.1145/3472674.3473981

[CR77] Pontillo V, Palomba F, Ferrucci F (2022) Static test flakiness prediction: how far can we go? - online appendix -. 10.6084/m9.figshare.17080946 10.1007/s10664-022-10227-1 PMC9526694 36199835

[CR78] Quinlan JR (1986). Induction of decision trees. Mach Learn.

[CR79] Qusef A, Bavota G, Oliveto R, Lucia AD, Binkley D (2013). Evaluating test-to-code traceability recovery methods through controlled experiments. J Softw Evol Process.

[CR80] Rehman MHU, Rigby PC (2021) Quantifying no-fault-found test failures to prioritize inspection of flaky tests at ericsson. In: 29Th ACM joint meeting on european software engineering conference and symposium on the foundations of software engineering, pp 1371–1380

[CR81] Schapire RE (2013) Explaining adaboost. In: Empirical inference, Springer, pp 37–52

[CR82] Shabtai A, Elovici Y, Rokach L (2012) A survey of data leakage detection and prevention solutions. Springer Science & Business Media

[CR83] Shannon CE (1948). A mathematical theory of communication. Bell Syst Tech J.

[CR84] Shi A, Lam W, Oei R, Xie T, Marinov D (2019) Ifixflakies: a framework for automatically fixing order-dependent flaky tests. In: ESEC/FSE 2019, pp 545–555

[CR85] Spadini D, Palomba F, Zaidman A, Bruntink M, Bacchelli A (2018) On the relation of test smells to software code quality. In: 2018 IEEE international conference on software maintenance and evolution (ICSME), IEEE, pp 1–12

[CR86] Taud H, Mas J (2018) Multilayer perceptron (mlp). In: Geomatic approaches for modeling land change scenarios, Springer, pp 451–455

[CR87] Terragni V, Salza P, Ferrucci F (2020) A container-based infrastructure for fuzzy-driven root causing of flaky tests. In: ICSE 2020, pp 69–72

[CR88] Thorve S, Sreshtha C, Meng N (2018) An empirical study of flaky tests in android apps. In: ICSME 2018, IEEE, pp 534–538

[CR89] Tufano M, Palomba F, Bavota G, Oliveto R, Di Penta M, De Lucia A, Poshyvanyk D (2017). When and why your code starts to smell bad (and whether the smells go away). IEEE Trans Softw Eng.

[CR90] van Deursen A, Moonen L, Van Den Bergh A, Kok G (2001) Refactoring test code. In: XP 2001, Citeseer, pp 92–95

[CR91] Van Rompaey B, Du Bois B, Demeyer S, Rieger M (2007). On the detection of test smells: a metrics-based approach for general fixture and eager test. IEEE Trans Softw Eng.

[CR92] Vassallo C, Panichella S, Palomba F, Proksch S, Gall HC, Zaidman A (2020). How developers engage with static analysis tools in different contexts. Empir Softw Eng.

[CR93] Vavrová N, Zaytsev V (2017) Does python smell like java? tool support for design defect discovery in python. arXiv:1703.10882

[CR94] Wang T, Golubev Y, Smirnov O, Li J, Bryksin T, Ahmed I (2021) Pynose: a test smell detector for python. In: 2021 36Th IEEE/ACM international conference on automated software engineering (ASE), IEEE, pp 593–605

[CR95] Webb GI, Keogh E, Miikkulainen R (2010). Naïve bayes. Encycl Mach Learn.

[CR96] Wong WE, Horgan JR, London S, Agrawal H (1997) A study of effective regression testing in practice. In: PROCEEDINGS the eighth international symposium on software reliability engineering, IEEE, pp 264–274

[CR97] Yen SJ, Lee YS (2006) Under-sampling approaches for improving prediction of the minority class in an imbalanced dataset. In: Intelligent control and automation, Springer, pp 731–740

[CR98] Zhang S, Jalali D, Wuttke J, Muṡlu K, Lam W, Ernst M, Notkin D (2014) Empirically revisiting the test independence assumption. In: ISSTA 2014, pp 385–396

[CR99] Zheng W, Liu G, Zhang M, Chen X, Zhao W (2021) Research progress of flaky tests. In: 2021 IEEE international conference on software analysis, evolution and reengineering (SANER), IEEE, pp 639–646

